# Induction of Synthetic Apomixis in Two Sorghum Hybrids Enables Seed Yield and Genotype Preservation Over Multiple Generations

**DOI:** 10.1111/pbi.70441

**Published:** 2025-11-05

**Authors:** Marissa K. Simon, Li Yuan, Ping Che, Kevin Day, Todd Jones, Ian D. Godwin, Anna M. G. Koltunow, Marc C. Albertsen

**Affiliations:** ^1^ Corteva Agriscience Johnston Iowa USA; ^2^ Centre for Crop Science, Queensland Alliance for Agriculture and Food Innovation The University of Queensland St Lucia Australia

**Keywords:** self‐reproducing hybrids, sorghum, synthetic apomixis

## Abstract

Induction of apomixis, or clonal reproduction through seed, could economise commercial hybrid seed production and enable smallholder farmers to save and sow hybrid seed. Here, we demonstrate the synthetic induction of apomixis in two sorghum hybrids and show that the clonal hybrid seed can be maintained across multiple seed generations. This was achieved through the combination of avoidance of meiosis and induced parthenogenesis. Meiotic avoidance was generated by CRISPR/Cas9 knockout of the sorghum meiosis genes *Spo11*, *Rec8*, *OsdL1* and *OsdL3*. Parthenogenesis was induced in the resultant diploid egg cell by the expression of the *Cenchrus ASGR‐BBML2* gene coding sequence. Two different strategies were used to combine these components to induce synthetic apomixis in two sorghum hybrids. Each hybrid used Tx623 as a female parent and either Tx430 or the African landrace Macia as a male parent. Seed yields in both apomictic hybrids were consistent and stable for multiple generations following self‐pollination but reduced relative to the sexual hybrids. Sorghum contains two copies of the *Osd1* gene that function in meiotic non‐reduction. CRISPR/Cas9 knockout of both *OsdL1* and *OsdL3* loci was sufficient to produce clonal hybrid progeny in conjunction with the other components, but this led to a reduction in seed set. By contrast, a single in‐frame edited allele of either *OsdL1* or *OsdL3* significantly improved seed set of clonal hybrid progeny. Fine‐tuning *OsdL* activity appears to be essential to optimising fertility; however, additional improvements are required to unlock the agronomic potential of synthetically induced apomictic sorghum in the field.

## 1 Introduction

Sexual reproduction in flowering plants leads to seed formation and is essential for the creation of genetic diversity through recombination during gametogenesis and subsequent parental gamete fusion during fertilisation. However, some species can form seed asexually via apomixis where the maternal genotype is preserved in the progeny (Nogler 1984). The developmental pathways resulting in apomictic seed vary in different apomictic species (Koltunow and Grossniklaus 2003). Although apomixis occurs naturally in hundreds of plant species, it is absent in most major crop species (Underwood and Mercier 2022).

Due to the ability of apomicts to produce seeds that retain the maternal genotype, apomixis has long been considered for applications in plant breeding (Hanna and Bashaw 1987). Introduction of apomixis from natural species to sexual relatives by plant breeding has generally proved inefficient or unsuccessful with respect to clonal seed production (Hanna and Bashaw 1987; Leblanc et al. 1996; Richards 2003). Engineered apomixis would be particularly valuable in naturally self‐pollinating species where large‐scale hybrid seed production is challenging. Its impact on the production of cross‐pollinated crops would economise hybrid seed production, and smallholder farmers would benefit from the availability of previously inaccessible hybrid seed.

Strategies have been borrowed from mechanisms in natural apomictic species for engineering apomixis into crop plants. Two broad categories of apomictic mechanisms are sporophytic and gametophytic apomixis (Hand and Koltunow 2014). Most efforts to date have focused on mimicking the mechanism of gametophytic apomixis where meiotic avoidance is combined with parthenogenesis to produce unrecombined, unreduced gametes and fertilisation‐independent embryo formation (Underwood and Mercier 2022). Meiotic avoidance, or apomeiosis, can be achieved through mutagenesis to induce the *Mitosis instead of Meiosis*, or *MiMe*, phenotype (d'Erfurth et al. 2009). *MiMe* was first implemented in Arabidopsis by combining the loss‐of‐function mutants *Atspo11‐1*, *Atrec8* and *Atosd1* (d'Erfurth et al. 2009). In *MiMe*, the *spo11* mutation disrupts homologous recombination, and when combined with the *rec8* mutation, functionally converts meiosis I into a mitosis‐like division with separation of sister chromatids without crossing over. Then, the *osd1* mutation functions to eliminate meiosis II (d'Erfurth et al. 2009), successfully replacing meiosis with mitosis. The replacement of meiosis with mitosis using orthologous gene mutations was subsequently achieved in rice (Mieulet et al. 2016).

The capability to induce parthenogenesis is possible by utilizing members of AP2 transcription factor genes, functionally referred to as the *BABYBOOM* (*BBM*) family, first shown to induce embryos and cotyledon‐like structures on seedlings in Arabidopsis (Boutilier et al. 2002). The natural apomict *Cenchrus squamulatus* (syn. *Pennisetum squamulatum*), utilises the apospory‐specific genomic region *BABYBOOM‐LIKE2* gene (*ASGR‐BBML2*) to induce parthenogenesis. The *ASGR‐BBML2* gene is expressed in the egg cell up to two days before anthesis leading to embryonic cell divisions and parthenogenetic embryo formation in the ovule (Conner et al. 2015). Furthermore, *ASGR‐BBML*2 directed to the egg cell can induce haploid embryo production in sexual pearl millet (Conner et al. 2015), rice, and maize (Conner et al. 2017), pointing to the utility of this gene as a component in engineering apomixis in cereal grains. *MATRILINEAL* (*MTL*)/*NOT LIKE DAD* (*NLD*)/*ZmPLA1* also has been identified as a locus responsible for haploid seed formation in maize (Gilles et al. 2017; Kelliher et al. 2017; Liu et al. 2017), and the *Taraxacum officinale PARTHENOGENESIS* (*ToPAR*) gene has been identified to induce haploid embryo‐like structures when expressed in lettuce (Underwood et al. 2022).

By combining Clustered Regularly Interspaced Short Palindromic Repeats/CRISPR‐associated protein 9 (CRISPR/Cas9) knockout of the *MiMe* components, together with parthenogenesis induced by either *BBM, MTL or ToPAR* expression, synthetic apomixis has been successfully engineered in rice (Dan et al. 2024; Huang et al. 2025; Khanday et al. 2019; Liu et al. 2023; Song, Li, et al. 2024; Vernet et al. 2022; Wang et al. 2019; Wei et al. 2023; Xie et al. 2019; Song, Wang, et al. 2024). In Arabidopsis, where parthenogenetic embryogenesis cannot be induced through *BBM* expression, clonal seeds have instead been engineered by combining *MiMe* with genome elimination after fertilisation (Marimuthu et al. 2011; Ravi and Chan 2010). While these studies prove that induction of synthetic apomixis is possible, it is evident that additional work is needed to improve the efficiency of clonal seed production to approach yields of sexually produced hybrids. Seed yield comparable to sexually produced hybrids and near 100% penetrance of clonal seed is required to create commercially viable hybrid seed products and to maximise on‐farm production (Heidemann et al. 2025; Underwood and Mercier 2022).

While synthetic apomixis has been successfully induced in rice and Arabidopsis, other plant species where clonal seed production could provide agronomic advantages have so far been unsuccessful at scale. Sorghum ( *Sorghum bicolor* ) is a cereal grain that was domesticated in sub‐Saharan Africa (de Wet 1978). It remains a subsistence food crop for millions of resource limited sub‐Saharan African smallholder farmers. It is also grown worldwide for human food, livestock feed, biofuel, and forage (Hossain et al. 2022; Khalifa and Eltahir 2023; Kazungu et al. 2023). Compared with other major cereal crops, sorghum has increased resilience and performance under stressful conditions and on marginal land (Khalifa and Eltahir 2023). Sorghum is naturally self‐pollinating, making hybrid seed production an under‐utilised source of genetic gain in resource‐limited areas of the world. Engineering self‐reproducing (SR) hybrids through apomixis would provide smallholder farmers with saveable, high‐yielding hybrid seed that harnesses heterosis. It would also economise hybrid sorghum production and advance new hybrid varieties for specific agroecological regions in both commercial and resource‐limited contexts.

Here we have demonstrated that the induction of *MiMe* via gene editing of endogenous sorghum genes *Spo11‐1*, *Rec8* and two *Osd1‐Like* loci in combination with parthenogenesis elicited by transgenic introduction of the *Cenchrus ASGR‐BBML2* gene induces apomictic reproduction in sorghum. Induction of SR hybrids in two genetic backgrounds using two different methods were followed through multiple generations and clearly demonstrate the inheritance of the hybrid genotype. We observed a significant reduction in seed number per plant compared to the wild‐type sexual hybrid, with clear evidence that *Osd1‐Like* gene dosage is one factor crucial to plant growth and seed set in induced apomictic sorghum hybrids.

## 2 Results

### 2.1 Validating *Mitosis Instead of Meiosis* ( *MiMe* ) Component Function in the Tx430 Variety of Sorghum

**FIGURE 1:**
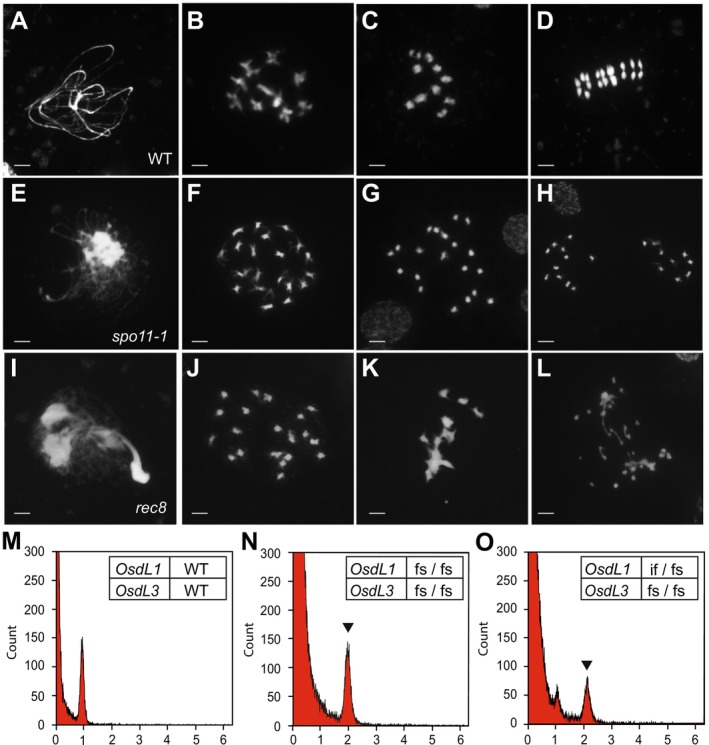
Sorghum male meiosis and pollen ploidy in wild type (WT) and *MiMe* component mutants. (A–D) WT Tx430 control, (E–H) *spo11‐1* mutant, (I–L) *rec8* mutant; at pachytene stage (A, E, I), diakinesis (B, F, J), early metaphase I (C, G, K), early anaphase I (D, H, L). Bars = 5 μm. (M–P) Flow cytometry histograms of propidium iodide stained nuclei isolated from pollen of WT control (M), and plants containing four frameshift (fs)*OsdL1*/*OsdL3* alleles (N), three frameshift and one in‐frame (if) *OsdL1*/*OsdL3* alleles (O). Arrowhead indicates diploid pollen nuclei (relative propidium iodide fluorescence *X*‐axis, number of nuclei *Y*‐axis).

The progression of meiosis in developing meiocytes in wild‐type sorghum are shown in Figure 1A–D, and mature tetrads are shown in Figure S1. *Spo11‐1* was targeted by CRISPR/Cas9 gene editing to prevent double strand breaks and chromosome pairing at meiosis I (Table S1). Cytology of male meiocytes in *spo11‐1* mutant plants (Figure 1E–H) showed modifications to meiosis with 20 univalents (Figure 1F,G) instead of 10 bivalents observed in wild‐type TX430 (Figure 1B,C). Random segregation of chromosomes occurred during meiosis I (Figure 1H), producing unbalanced tetrads, polyads and triads (Figure S1) at the completion of meiosis II. As expected, the *spo11‐1* mutant plants were almost completely sterile following self‐pollination.

*Rec8* was targeted for CRISPR/Cas9 knockout to prevent sister chromatid cohesion at meiosis I (Table S1). Cytology of male meiocytes in *rec8* mutant plants (Figure 1I–L) showed reduced homolog pairing and 20 univalents (Figure 1I,J) instead of 10 bivalents (Figure 1B,C). Early sister chromatid segregation and chromosome fragmentation occurred during meiosis I (Figure 1K,L) and polyads were evident at completion of meiosis II (Figure S1). Self‐pollinated *rec8* mutant plants were completely sterile and did not produce any seed.

Sorghum contains two tightly linked *Osd1‐Like* genes (*OsdL1* and *OsdL3*) separated by 7802 bp rather than the single *Osd1* gene identified and functionally characterised in rice (Mieulet et al. 2016). A third *Osd1*‐*Like* gene identified in sorghum is outside the *Osd1* clade identified in rice (Mieulet et al. 2016). The function of the *OsdL1* and *OsdL3* genes in the progression of meiosis was examined by individually targeting each gene for knockout with CRISPR/Cas9 (Table S1). *Osd* mutations are expected to produce unreduced diploid gametes, and when self‐fertilised, these mutants should generate tetraploid progeny. Homozygous knockouts in either *OsdL1* or *OsdL3* however, only produced diploid progeny (Table 1). Crossing of *OsdL1* and *OsdL3* single gene edits to produce heterozygous alleles linked in *trans* also produced diploid progeny (Table 1).

**TABLE 1:** Examination of the ploidy of progeny in Tx430 sorghum transgenics containing mutational edits in *OsdL1* and *OsdL3* genes.

Edit	*OsdL1* allele	*OsdL3* allele	Progeny tested	Diploid	Tetraploid
*OsdL1* single edit	−1232/−1232	WT	32	32	0
*OsdL3* single edit	WT	−1537/−1537	33	33	0
Crossing *OsdL* single edits	−1232/WT	−1537/WT	32	32	0
*OsdL1*/*OsdL3* double edit	−30/−30	−11/−9	12	11	1
	−11/−11	−1/−3	41	1	40
	+1/−15	−1/−23	49	0	49
	+1/+1	−1/−1	28	0	28

To examine the possibility of functional redundancy, both *OsdL1* and *OsdL3* genes were targeted with CRISPR/Cas9 to simultaneously knockout gene function (Table S1). Double knockout mutants were obtained with four frameshift mutations. In addition, plants with three frameshift mutations and one in‐frame mutation in either of the *OsdL1* or the *OsdL3* genes were also obtained. Pollen flow cytometry analyses (Figure 1M–O) showed that haploid pollen was produced in wild‐type Tx430 (Figure 1M), whereas *osdL1 osdL3* double knockout plants produced diploid pollen (Figure 1N). Plants containing three frameshift alleles and one in‐frame allele produced both haploid and diploid pollen (Figure 1O).

To characterise the combined effect of the above mutations on male and female gamete non‐reduction, we measured ploidy levels of the progeny. All progeny from the double knockout plants were tetraploid (Table 1). Progeny from a parent that contained three frameshift alleles and one in‐frame allele were mostly tetraploid, whereas progeny from a parent containing one in‐frame allele and three frameshift alleles were mostly diploid (Table 1). Collectively, these data indicate that the *OsdL1* and *OsdL3* genes are functionally redundant, and that mutations in both genes are required to give a fully penetrant non‐reduction phenotype.

### 2.2 Parthenogenesis Induction in the Sorghum Variety Tx430

Six constructs were developed to examine parthenogenesis induction in sorghum (Table S2). Two constructs contained the previously identified apomictic *Cenchrus ASGR‐BBML2* promoter combined with either the *ASGR‐BBML2* genomic (gDNA) sequence or the coding (cDNA) gene sequence (Conner et al. 2017). The expression pattern of the *ASGR‐BBML2* promoter was unknown in sorghum, therefore, an additional four constructs were developed that contained the maize orthologous promoters of the originally identified Arabidopsis *DD45* and *RKD2* egg cell preferred expressed genes (Albertsen et al. 2013), fused to the *Cenchrus ASGR‐BBML2* genomic or coding gene sequences (Conner et al. 2017).

**FIGURE 2:**
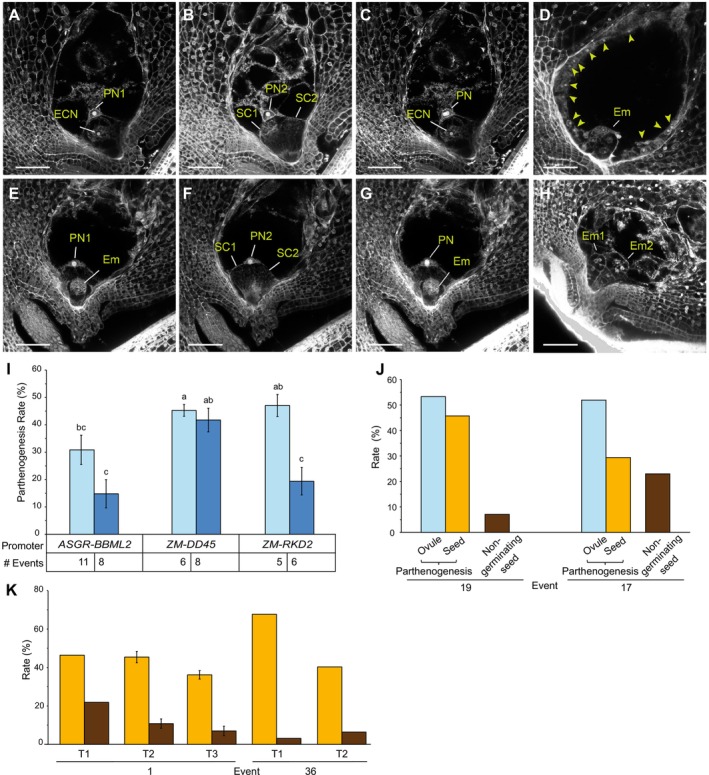
Cytological and progeny analyses of induced parthenogenesis in sorghum. (A–D) Emasculated and fertilised female gametophytes in wild type (WT) sorghum. Two focal planes (A, B) with the merged image (C) of an emasculated embryo sac. Fertilised embryo sac, 2 days after pollination, with a fertilised embryo and syncytial endosperm nuclei (arrowheads) (D). E–H show emasculated female gametophytes in parthenogenetic sorghum. Two focal planes (E, F) with the merged image (G) of an emasculated embryo sac with parthenogenesis. Two parthenogenetic embryos in a single gametophyte (H). ECN, egg cell nucleus; SC, synergid cell; PN, polar nucleus; Em, embryo. Bars = 45 μm. (I) Mean parthenogenesis rate ± SE in transformants containing *ASGR‐BBML2* gDNA (light blue) and cDNA sequences (dark blue) driven by three different promoters (*ASGR‐BBML2*, maize *DD45* and maize *RKD2* promoters). Number of events per construct are indicated. Constructs with significantly different rates are indicated with different letters (Student's t‐test, two tailed *p* > 0.05). (J) Comparison of parthenogenesis rate determined by cytological analysis (light blue), progeny flow cytometry (orange), and seed non‐germination rates (brown) observed in events 19 and 17 of *ASGR‐BBML2 pro:ASGR‐BBML2* (PHP83000). (K) Comparison of parthenogenesis rate ± se, as determined by flow cytometry (orange) and seed non‐germination rate (brown) of events 1 and 36 of *ZM‐DD45 pro:ASGR‐BBML2* cDNA (PHP94292) in T1, T2 and T3 generations.

Parthenogenetic events were evaluated by cytological observations of embryo development in the female embryo sac post‐anthesis in wild‐type and transgenic plants. Unpollinated wild‐type Tx430 sorghum results in the female gametophyte remaining unfertilised with a single egg cell and two unfused polar nuclei (Figure 2A–C). After pollination, double fertilisation of the female gametophyte results in the embryo developing after egg cell fusion with one sperm cell, and the endosperm developing after fusion of both polar nuclei with the second sperm cell (Figure 2D). In a hemizygous sexual transgenic plant containing an egg cell expressed *ASGR‐BBML2* transgene to induce parthenogenesis, 50% of the female gametophytes will inherit the transgene with the egg cell initiating parthenogenetic embryo development and the polar nuclei remaining unfused.

Transformation of the six constructs into Tx430 produced a range of 5–11 T0 plants for each construct. A total of 44 unfertilised hemizygous *ASGR*‐*BBML2* transgenic T0 plants were cytologically examined for parthenogenesis as determined by the development of an embryo‐like structure observed at the micropylar end of the embryo sac, together with the presence of two unfused polar nuclei, and the corresponding absence of endosperm development (Figure 2E–G,I, Table S3). The development of two parthenogenetic embryos was observed in female gametophytes in 0.9% of ovules examined (Figure 2H). Parthenogenesis was induced by all six constructs (Figure 2I). Three of the six constructs, containing the maize *DD45* promoter fused to the *ASGR‐BBML2* gDNA or cDNA, or the maize *RKD2* promoter fused to the *ASGR‐BBML2* gDNA, induced average parthenogenesis rates greater than 40% (Figure 2I, Table S3). The other three constructs, containing the native *ASGR‐BBML2* promoter fused to the *ASGR‐BBML2* gDNA or cDNA, or the maize *RKD2* promoter fused to the *ASGR‐BBML2* cDNA, induced average parthenogenesis rates that ranged from 10% to 30% (Figure 2I, Table S3). The maize *DD45* promoter induced parthenogenesis at equivalent rates when fused to both the *ASGR*‐*BBML2* gDNA and cDNA sequences. The native *ASGR‐BBML2* and the maize *RKD2* promoters were less efficient in stimulating parthenogenesis when linked to the *ASGR‐BBML2* cDNA relative to the gDNA.

Progeny from two selected T0 events (17 and 19), containing the native *ASGR‐BBML2* promoter fused to the *ASGR*‐*BBML2* gDNA that showed high rates of parthenogenesis in cytological evaluations, were further examined by cytology and flow cytometry to examine rates of embryo initiation and germination of seeds containing haploid embryos. In event 19, the cytology and flow cytometry showed similar rates of haploid embryo initiation and haploid seed development, but with 6.3% non‐germinated seed. In event 17, the frequency of haploid embryo initiation was much greater than the observed haploid seed development, with 20.3% non‐germinated seed (Figure 2J). These results suggest that position effects of the introduced transgene into the genome may also influence the maturation of haploid embryos in seeds.

For the forward combination of parthenogenesis induction with *MiMe*, another parthenogenesis construct was designed to generate transgenic lines containing the maize *DD45* promoter and *Cenchrus ASGR‐BBML2* cDNA sequence that had demonstrated good penetrance of the parthenogenesis phenotype (Figure 2I). This construct was designed to produce plants that would not contain additional T‐DNA components (Table S2). An advantage of this construct design is that it should eliminate the effect of adjacent promoters in the T‐DNA from potentially influencing the expression pattern or level of the parthenogenesis cassette. As shown in Figure 2K, flow cytometry of two transgenic events containing this construct showed a high frequency of haploid progeny across multiple generations. Two embryos in a single seed were observed in 3.0% of progeny seed across both events. Germination and subsequent ploidy determination of these dual embryo progeny seed revealed that most dual embryos were haploid‐haploid pairs and rarely haploid‐diploid pairs (data not shown). It is unknown if the dual haploid embryos originate by embryo budding, possible synergid embryo induction, or if the diploids in the haploid pairs arise from sexual fertilisation or by spontaneous doubling as occurs in some species (Fomicheva et al. 2024).

### 2.3 Two‐Step Induction of Synthetic Apomixis in a Tx623/Tx430 Hybrid

**FIGURE 3:**
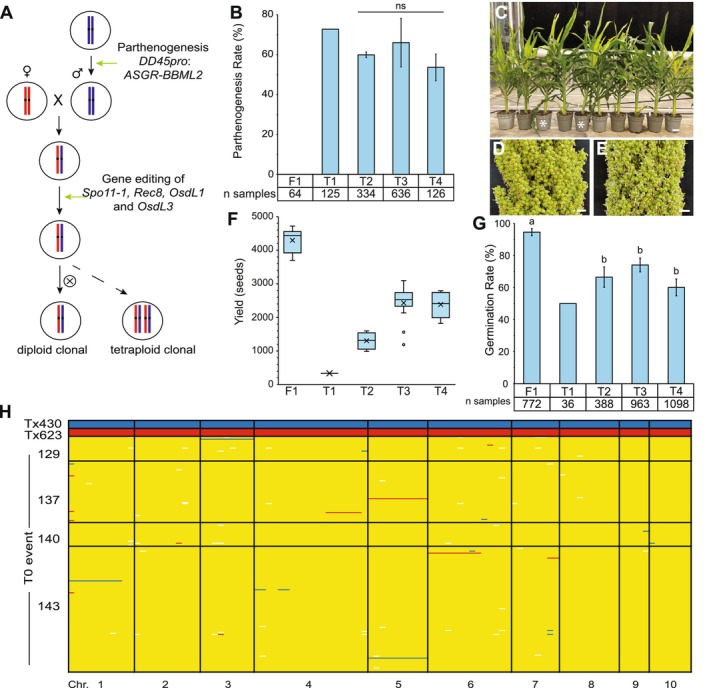
Analyses of synthetic apomixis in Tx623/Tx430 hybrid sorghum induced by a two‐step approach. (A) Schematic representation of two‐step method to induce synthetic apomixis. (B) Mean parthenogenesis rate (±SE), of the sexual F1 hybrid control, and T1, T2, T3 and T4 generations derived from transgenic event 143, with sample numbers for each generation shown. Parthenogenesis rates from T2, T3 and T4 were not significantly (ns) different (single factor ANOVA, *p* = 0.654). (C–E) Comparison of plant and panicle morphology. (C) Whole plant phenotype of synthetic hybrid apomicts and F1 sexual hybrid controls (indicated with *). Bar = 10 cm. Panicle of F1 sexual hybrid (D), and apomictic T2 plant (E). Bars = 1 cm. (F) Box plots of seed yields per from the F1 hybrid control and T1, T2, T3 and T4 generations of event 143. (G) Mean seed germination rates (±SE), of a F1 hybrid control and T1, T2, T3 and T4 generations of event 143, with total samples for each population shown. Significantly different germination rates are indicated with different letters (Student's *t*‐test, two‐tailed *p* < 0.05). (H) SNP marker analysis of the Tx623 and Tx430 parental controls and 238 T1 progeny from four selected T0 events. The Tx430 alleles are marked in blue, Tx623 alleles in red, heterozygous alleles in yellow, missing marker calls in white, and chromosome (chr) is indicated.

Two different approaches were used to combine *MiMe* with parthenogenesis to produce sorghum SR hybrids. A two‐step approach was used in the generation of SR Tx623/Tx430 sorghum hybrids. In the first step, a characterised Tx430 transgenic line with the maize *DD45* promoter*:ASGR‐BBML2* cDNA was crossed with a wild‐type Tx623 sorghum line to generate a F1 hybrid. In the second step, immature embryos from this cross were used in a secondary transformation to introduce CRISPR/Cas9 together with *MiMe* guide RNAs (gRNA) (Figure 3A). The frequency of editing at each individual *MiMe* locus was consistent and ranged from 45.3% to 47.3% (Table S4). In total, 33 plants were retained with a hemizygous *ASGR‐BBML2* transgene and a combination of unique edits at the *MiMe* loci. T0 biallelic edits were germinal and were clonally inherited in T1 progeny (Table S5).

A range of phenotypes were observed in T0 plants in the greenhouse, including some plants that showed significantly delayed and aberrant development with a reduction in plant height, leaf and stem curling, and reduced seed set (Figure S2). It is noteworthy that seed number per plant was significantly reduced in the T0 apomictic hybrids with complete knockouts of all four *OsdL1* and *OsdL3* alleles (Table 2). There was, however, a clear improvement in the fertility of T0 plants that contained three frameshift alleles and one in‐frame allele in the *OsdL1* and *OsdL3* loci relative to T0 plants that contained *OsdL1* and *OsdL3* loci with four frameshift alleles (Table 2). Four independent T0 events were chosen for further characterisation of apomictic progeny in the T1 generation relative to a hybrid control. Multiple progeny from each event, however, showed reduced seed set in comparison to the sexual hybrid control (Table 3).

**TABLE 2:** Effect of *OsdL1* and *OsdL3* gene dosage on T1 seed production in Tx623/Tx430 and Tx623/Macia synthetic apomictic plants (fs indicates a frameshift allele).

Hybrid	Apomixis induction	*n*	Average ± SD seed count	*OsdL* allele edit types	*n*	Average ± SD seed count
Tx623/Tx430	Two‐step method (PHP94292 + PHP103035)	33	210.3 ± 520.7	4 fs alleles	21	36.6 ± 91
				3 fs alleles + 1 in‐frame allele	12	440.7 ± 784.4
Tx623/Macia	One‐step method (PHP105181)	44	433.6 ± 824.9	4 fs alleles	9	12 ± 27.2
				3 fs alleles + 1 in‐frame allele	27	655.1 ± 983.6
				2 fs allele + 2 in‐frame alleles	7	89.9 ± 189.9

**TABLE 3:** Analysis of progeny ploidy and seed set in four representative apomictic events in Tx623/Tx430 and Tx623/Macia hybrids.

Hybrid	Apomixis induction	T0 event	T1 seed count	% T1 diploid frequency	T2 seed count average ± SD (*n* plants)
Tx623/Tx430	Two‐step method (PHP94292 + PHP103035)	140	202	87.5	68.5 ± 61.6 (6)
		143	201	74.8	1264.8 ± 222.5 (4)
		137	449	82.3	300.8 ± 258.4 (13)
		129	491	68.0	601.7 ± 382.5 (3)
	F1 control	n/a	n/a	n/a	4222 ± 329.7 (6)
Tx623/Macia	One‐step method (PHP105181)	105	559	48.9	n.t.
		120	3533	29.9	1488.7 ± 537.8 (6)
		60	1383	53.8	195 ± 11.3 (2)
		78	2042	57.5	n.t.
	F1 control	n/a	n/a	n/a	3860 ± 1678 (15)

*Note:* T1 seed count is the number of seeds produced on a T0 plant, and T2 seed count is the number of seeds produced on a T1 plant.

Abbreviation: n.t., not tested.

The Tx430 *ASGR‐BBML2* parthenogenesis event produced 43% haploid progeny when hemizygous in the sexual parent plant (Figure 2K). When this parthenogenesis event was combined with *MiMe* mutations with three frameshift alleles within both *OsdL* genes and one in‐frame *OsdL* allele in either gene, the expected progeny ratios were 86% parthenogenetic diploids and 14% non‐parthenogenetic tetraploids. The observed frequency of diploid progeny ranged from 68.0% to 87.5% across the four independent events (Table 3, Figure S3).

To confirm the successful production of synthetic apomictic clonal sorghum progeny, 238 T1 progeny that were produced from four independent T0 events, including 184 diploid plants and 54 tetraploid plants, were evaluated with single‐nucleotide polymorphism (SNP) markers. Over 96% of the analysed T1 progeny (229 of the 238 plants) were heterozygous on all chromosomes (Figure 3H). Therefore, the identification of hybrid diploid clonal progeny demonstrates that the combination of *MiMe* and *ASGR‐BBML2* was successful in the female gamete to induce synthetic apomixis. The identification of hybrid tetraploid progeny demonstrates that *MiMe* was active in both the male and female gametes. Unexpectedly, nine individual progeny showed a SNP allele pattern that suggested these were aneuploid. Among these aneuploid progeny, three plants had lost one copy of a single chromosome, and six plants had a partial deletion of a single chromosome (Figure 3H).

Transgenic event 143, containing one in‐frame allele in *OsdL1* and three frameshift alleles in *OsdL1* and *OsdL3*, that showed good seed set in the T1 generation was selected for further analysis in subsequent T2, T3 and T4 generations. The relative parthenogenesis frequency as measured by the frequency of diploid plants in the T1, T2, T3 and T4 generations of event 143 was stable across generations (Figure 3B). The maintenance of heterozygosity in both the diploid and tetraploid progeny was evaluated in each generation using the same set of SNP markers described above. The *MiMe* phenotype was stable and highly penetrant in progeny of event 143 from T2, T3 and T4 generations (*n* = 207, 638 and 126, respectively). Similar to the results observed in the T1 generation, over 98% of the T2 progeny, 93% of the T3 progeny and 97% of the T4 progeny had SNP markers showing heterozygous alleles across all chromosomes (Figure S4). The remaining plants in each generation were aneuploid with either a large deletion of a single chromosomal segment or a deletion of an entire single chromosome.

In addition to molecular analyses of event 143, plant phenotype observations of vegetative development, panicle length and initial pollen shed date showed no obvious differences between apomictic plants and the hybrid controls (Figure 3C, Figure S5). Differences were noted, however, during panicle development where many florets did not show evidence of seed development, which correlated with reduced seed set (Figure 3D–F). The relative reduction of seed set was consistent across multiple generations (Figure 3F). Seeds were phenotypically normal (Figure S6), although both 1000‐grain weight and seed germination of the apomictic hybrids was reduced relative to the hybrid controls (Figure S5, Figure 3G). Flow cytometry showed that the ploidy of the endosperm of the synthetic apomictic lines was increased to 6n, compared to 3n endosperm in the sexual control (Figure S7). This is expected given the presence of two diploid central cell nuclei fertilised by a diploid sperm cell nucleus. Collectively, these data show that SR hybrid sorghum can be generated in a two‐step induction approach, and that induced synthetic apomictic seed set is maintained over at least four generations.

### 2.4 One‐Step Induction of Synthetic Apomixis in Sorghum in a Tx623/Macia Hybrid

**FIGURE 4:**
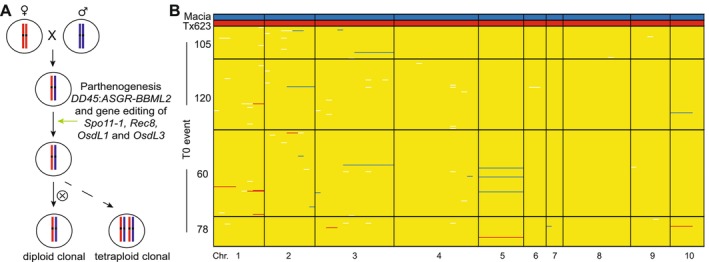
Generation of synthetic apomixis in Tx623/Macia hybrid sorghum induced by a one‐step approach. (A) Schematic representation of one‐step induction of synthetic apomixis. (B) SNP marker analysis of the Tx623 and Macia parental controls and 295 T1 progeny from four selected T0 events. The Macia alleles are marked in blue, Tx623 alleles in red, heterozygous alleles in yellow, missing marker calls in white, and chromosome (chr) is indicated.

A second approach to induce synthetic apomixis in sorghum in a single step was conducted in a hybrid cross between Tx623 as the female parent and the African variety Macia. Immature embryos from the F1 hybrid cross were used in a primary transformation to introduce the maize *DD45* promoter*:ASGR‐BBML2* cDNA parthenogenesis cassette together with CRISPR/Cas9 and *MiMe* gRNAs in a single T‐DNA construct (Figure 4A). The efficiency of CRISPR/Cas9 mediated mutations at each of the four target loci was evaluated by amplicon sequencing and was found to be consistent, ranging from 51.3% to 53.1% (Table S4). In total, 44 plants were retained and analysed for *MiMe* loci edits (Table S5) and for inheritance of *ASGR‐BBML2*. Similar to the two‐step synthetic apomixis experiment, it was clear that there was a relative improvement in the overall reproductive fertility in the T0 plants that contained three frameshift alleles and one in‐frame allele in the *OsdL1* and *OsdL3* loci relative to the T0 plants that contained four frameshift alleles (Table 2).

Four independent T0 events were chosen for further characterisation of apomictic T1 progeny relative to a hybrid control. Seed set of the T1 plants was reduced and variable relative to the hybrid control (Table 3). Parthenogenesis frequencies in T1 progeny of these selected apomictic events ranged from 29.9% to 57.5% (Table 3, Figure S3). In total, 295 T1 progeny were analysed for heterozygosity, including 137 diploid plants and 158 tetraploid plants. Similar to the *MiMe* results shown above for the Tx623/Tx430 hybrid, over 96% of the T1 progeny analysed were clonal (284 of the 295 plants), with heterozygous alleles across all chromosomes (Figure 4B). Only 11 plants showed large or entire chromosomal deletions that suggested the production of aneuploid progeny (Figure 4B). These data indicated that SR hybrid sorghum can also be generated when all of the induction components are introduced within one T‐DNA plasmid.

## 3 Discussion

This is the first report of synthetic apomixis induction in hybrid sorghum and demonstration that the resultant self‐reproducing hybrids maintain heterozygosity in multiple self‐fertilised progeny generations. Introduction of synthetic apomixis into hybrid sorghum was successful by combining CRISPR/Cas9 induced mutations in the *MiMe* loci together with an egg cell expressed *ASGR‐BBML2* transgenic component. Importantly, synthetic apomixis was demonstrated in two hybrid backgrounds using two different induction approaches (one‐step and two‐step integration techniques). The two‐step approach has the advantage of first producing stable parthenogenesis phenotypes in all progeny, whereas the one‐step approach minimises the number of transformation steps, although it produces more variability in parthenogenesis.

Contrary to previous reports in rice (Khanday et al. 2019; Vernet et al. 2022) that showed an improvement in the induction of parthenogenesis and resulting synthetic apomictic progeny when using a one‐step technique, in sorghum we observed greater variability in parthenogenesis with a similar approach. Nevertheless, both approaches offer unique advantages to implement apomixis. Identifying a construct design that consistently delivers high rates of parthenogenesis is essential.

We demonstrated parthenogenesis induction rates of approximately 40% in multiple independent transgenic lines and showed that the use of a maize egg cell *DD45* promoter with the *Cenchrus ASGR‐BBML2* cDNA induced a more penetrant parthenogenesis phenotype than the *Cenchrus ASGR‐BBML2* promoter in sorghum. In addition, the observed event to event variation of parthenogenesis suggested that somaclonal variation associated with the transformation process or positional effects of the transgene integration site may influence the penetrance and expressivity of the parthenogenesis phenotype. Furthermore, our observation of the development and ploidy of dual embryos may highlight the importance of the developmental timing and specificity of the egg cell promoter driving the *ASGR‐BBML2* parthenogenesis gene.

Progeny seed germination was reduced in some transgenic sexual lines containing only the maize *DD45* promoter:*ASGR*‐*BBML2* cDNA construct. This suggests that the initiation of embryogenesis through parthenogenesis may have additional deleterious effects on the development of the embryo and seed. Improvement of parthenogenesis induction may benefit from further refinement of egg cell specific expression of *ASGR*‐*BBML2*, as well as the addition of other parthenogenesis genes that may supplement *ASGR‐BBML2* activity (Ren et al. 2024; Underwood et al. 2022; Huang et al. 2025).

In both synthetic apomixis experiments, we observed 96.2% of all SR hybrid progeny maintained complete heterozygosity. In the two‐step apomictic approach, incomplete penetrance of parthenogenesis resulted in 77.3% diploid and 22.7% tetraploid progeny. Thus, 73.9% of the total progeny were heterozygous diploid clonal progeny. In the one‐step apomictic approach, penetrance of parthenogenesis was more variable with 46.4% diploid and 53.6% tetraploid progeny, resulting in 43.4% of the total progeny as clonal progeny. Finally, the remaining 3.8% of all progeny from both experiments showed evidence of aneuploidy. Marker analysis of these plants showed a large, contiguous deletion of either a portion of a chromosome or of an entire chromosome, suggesting that at a low frequency chromosome fragmentation or mis‐segregation may be occurring in this *MiMe* system (*spo11‐1*, *rec8*, *osdL1*, *osdL3*). Similarly, previous studies have also identified the production of aneuploid progeny in *MiMe*‐2 (*spo11‐1, rec8, tam*) genotypes in *Arabidopsis* and tomato (d'Erfurth et al. 2009; Wang et al. 2024; Liu et al. 2023).

In Arabidopsis and rice, mutation of the single *Osd1* gene is sufficient to promote meiotic non‐reduction, resulting in unreduced male and female gametes (d'Erfurth et al. 2009; Mieulet et al. 2016). However, the incomplete penetrance of the non‐reduction phenotype resulted in reduced seed set due to male and female gamete ploidy imbalance at fertilisation (d'Erfurth et al. 2009; Mieulet et al. 2016). In sorghum we demonstrated that two tightly linked *Osd1‐Like* genes function in meiotic reduction. Analyses of four frameshift allele *osdL1* and *osdL3* mutants showed high penetrance of the non‐reduction phenotype in the male gametes, but this allelic combination unexpectedly produced significantly less seed in the synthetic apomicts compared with the sexual hybrid control. Incomplete non‐reduction in the female gametes may be a limiting factor influencing fertilisation and development of progeny seed. Detailed cytological characterisation of female meiosis may help assess if *OsdL1* and *OsdL3* functions equivalently in male and female meiosis.

Sorghum *OsdL1* and *OsdL3* may be essential for additional non‐meiotic cell cycle functions. In Arabidopsis, both *OSD1*/*GIG* and the related gene *UVI4* have been shown to affect mitotic cell cycle regulation (Iwata et al. 2011, 2012). Our analyses of sorghum *osdL1* and *osdL3* mutant combinations containing three frameshift alleles and one in‐frame allele showed an incomplete non‐reduction phenotype. However, this allelic combination unexpectedly led to improved seed set in apomicts compared to the four frameshift allelic combination, suggesting that the functional dosage of *OsdL* genes is important for production of viable gametes and subsequent viable progeny. The unique complement of edited alleles produced across events did not enable a complete evaluation of the full or partial functional redundancy of the *OsdL1* and *OsdL3* genes. Interestingly, the non‐reduction component of *MiMe* was also identified as a key step for further investigation in characterisation of *MiMe* genotypes in tomato (Wang et al. 2024). Therefore, optimal meiotic non‐reduction appears to be a critical component in enabling full fertility in synthetic apomictic systems.

The level of synthetic apomictic seed set observed in sorghum was, however, not equivalent to that of hybrid seed set in sexual controls indicating other factors are required to achieve efficient seed set. Similarly, several studies in rice have reported reduced fertility of synthetic apomictic lines (Dan et al. 2024; Huang et al. 2025; Khanday et al. 2019; Song, Li, et al. 2024; Vernet et al. 2022; Wang et al. 2019). Each of the component factors inducing synthetic apomixis in both rice and sorghum, modifies aspects of embryo and endosperm development producing a diploid embryo and a hexaploid endosperm instead of the typical monocot triploid persistent seed endosperm, which may or may not influence seed development, germination and seed quality. In this study we identified that both the choice of promoter to drive *ASGR*‐*BBML2* expression for optimal parthenogenesis and embryo development and the *OsdL* dosage for meiotic non‐reduction are critical factors for optimal induction of synthetic apomixis.

In conclusion, we have shown that sorghum is a crop in which synthetic apomixis is a reality. Clonal, diploid progeny from hybrid apomictic sorghum develops similarly to hybrid controls during vegetative growth. Seed set is reduced, but stable, across generations relative to a sexual hybrid control. While additional improvements in fertility will be required for commercial grain production, this technology has the potential to lead to significant improvements in agriculture by capturing heterosis and maintaining hybrid vigour through seed in sorghum. Resolving inefficiencies in this process are an important step for enabling field production of apomictic hybrids in sorghum.

## 4 Materials and Methods

### 4.1 Plant Materials and Handling

Sorghum genotypes Tx430, Tx623 and the African landrace Macia (PI 565121) were requested from USDA GRIN and were cultivated in the greenhouse with 25.5°C during the day and 20°C night temperatures under a 16 h photoperiod. To produce F1 embryo donor material for sorghum transformation for the two‐step approach, individual crosses were performed using a cytoplasmic male sterile (CMS) Tx623 (ATx623) as a female parent with the transgenic Tx430 (PHP94292 event 1) parthenogenesis line as the male parent. To produce F1 embryo donor material for sorghum transformation for the one‐step approach, individual crosses were performed using CMS sterile Tx623 (ATx623) as a female parent with the non‐transgenic Macia line as the male parent. Furthermore, fertile F1 progeny control seed of these two hybrids was produced by crossing the CMS sterile ATx623 as a female parent with either the non‐transgenic Tx430 or non‐transgenic Macia lines as the male parent. Crosses of Tx430 CRISPR/Cas9 edited lines were completed using hot water emasculation of the female parent immature panicle.

### 4.2 Construct Design

Seven plasmids were used for sorghum transformation to test parthenogenesis (Table S2). The *Cenchrus ASGR‐BBML2* promoter, *ASGR‐BBML2* genomic and *ASGR‐BBML2* coding sequence was generously provided by Peggy Ozias‐Akins (University of Georgia, USA). PHP83000 and PHP82772, contained the *Cenchrus ASGR‐BBML2* promoter driving the genomic or coding sequence of the *ASGR‐BBML2* gene, respectively, and a phosphomannose‐isomerase (*PMI*) selectable marker. PHP86483 and PHP86482 contained the maize *DD45* promoter, and PHP86484 and PHP86796 contained the maize *RKD2* promoter (Albertsen et al. 2013), each driving the genomic or coding sequence of the *ASGR‐BBML2* gene, respectively, and an herbicide‐resistant acetolactate synthase (*HRA*) selectable marker. A final plasmid, PHP94292, contained the same maize *DD45:ASGR‐BBML2* cassette as PHP86482.

Six plasmids were used for sorghum transformation to test gene editing of *MiMe* gene loci (Table S2). Plasmids PHP85484, PHP83595, PHP83596, PHP83436 each contained two gRNA cassettes targeting each sorghum gene *Spo11‐1*, *Rec8*, *OsdL1*, *OsdL3*, respectively, a maize optimised *Cas9* cassette, and a *PMI* selectable marker. Plasmids PHP85930 and PHP110251 contained two gRNA cassettes targeting the *OsdL1* and *OsdL3* genes, a maize optimised *Cas9* cassette and either a *PMI* or neomycin phosphotransferase II (*NPTII*) selectable marker.

Two plasmids were used for sorghum transformation to test the combination of *MiMe* editing and parthenogenesis (Table S2). For *MiMe* editing tests, plasmid PHP103035 contained a *Cas9* cassette, four gRNA cassettes (one gRNA for each *MiMe* target gene, two gRNAs driven by a maize *U6* promoter and two gRNAs driven by a sorghum *U6* promoter; Massel et al. 2022), and the *NPTII* selectable marker. For a combined parthenogenesis and gene editing test, plasmid PHP105181 contained the maize *DD45:ASGR‐BBML2* cassette, *Cas9* cassette, four gRNA cassettes, and the *NPTII* selectable marker.

### 4.3 Sorghum Transformation and Transgenic Edited Event Analyses

Immature embryo explants isolated from sorghum plants were transformed with *Agrobacterium* auxotrophic strain LBA4404 Thy‐ carrying a ternary vector transformation system to generate transgenic sorghum plants (Anand et al. 2018; Che et al. 2018). Conventional, morphogenic gene‐mediated, *Wus2*/*CRE*‐mediated marker‐free, and altruistic transformation methods were performed as previously described (Wu et al. 2014; Che et al. 2018, 2022). The integrated copy number of the T‐DNA and the vector backbone in these transgenic plants were determined by a series of qPCR analyses (Wu et al. 2014; Zhi et al. 2015). Amplicon deep sequencing to characterise CRISPR/Cas edits were performed as described in Che et al. (2022). Sequence reads were aligned to the Tx430, Tx623 or Macia wild‐type reference sequence, as appropriate, using Bowtie2. The gRNA target sites, sequences, and corresponding PAM for *Spo111*, *Rec8*, *OsdL1*, and *OsdL3* genes are shown in Figure S8. The primers used to amplify Sb‐*Spo11*, Sb‐*Rec8*, Sb‐*OsdL1* and Sb‐*OsdL3* genomic loci for amplicon analysis are shown in Table S6.

### 4.4 Cytological Analyses

Sorghum reproductive tissues were staged and collected before and after fertilisation from sorghum plants. For analysis of parthenogenetic embryo development, fertilisation was prevented by removing the stigmas from the immature ovules before anthesis and then ovules were collected three days after the stigma removal. A range of 15–25 ovaries from each plant were dissected using a LEICA MZ6 dissection microscope and then fixed in Carnoy's fixative solution (3:1 ethanol:acetic acid) for 1 day at 4°C. Ovaries were cleared in 100% methyl salicylate for at least one day. Cleared ovaries were observed under a LEICA DM5500 Q confocal microscope. Images were captured by LAS X software and further processed in Adobe Photoshop software. Male and female meiotic chromosome spreading was performed on meiotic stage floral buds fixed in Carnoy's fixative solution for one day at 4°C and prepared as described previously (Ross et al. 1996). Chromosomes were stained with DAPI (4,6‐diamino‐2‐phenylindole dihydrochloride; 1.5 μg/mL) (Vector Laboratories Inc, Burlingame, CA, USA) and observed with a LEICA DMRXA epifluorescence microscope system. Images were captured with LAS software and processed with Adobe Photoshop. Meiocytes were staged as described previously (Ross et al. 1996).

### 4.5 Flow Cytometry

Plant ploidy levels were measured using flow cytometry to detect nuclear DNA content. Approximately 16 mm^2^ fresh sorghum leaf tissue or pollen was added to a modified LB01 lysis buffer on ice (15 mM Tris, 2 mM Na_2_EDTA, 0.5 mM spermine 4HCl, 80 mM KCl, 20 mM NaCl, 0.1% (v/v) Triton X‐100, pH 7.5, 12.4 μM propidium iodide added before use, modified from prior studies; Doležel and Bartos 2005; Doležel et al. 1989), and was either chopped or lysed before being filtered through a 20 μm filter. Nuclei suspensions were analysed by the Attune NxT Flow Cytometer and the corresponding Attune cytometric software (v5.3.0).

### 4.6 SNP Marker Analysis

SNP marker genotyping was performed using TaqMan SNP genotyping assays (ThermoFisher) using sorghum leaf samples as described previously (Ye et al. 2024). A set of 104 and 87 polymorphic SNP markers covering all 10 sorghum chromosomes were used to evaluate Tx623/Tx430 and Tx623/Macia hybrid controls and self‐reproducing hybrid progeny, respectively.

### 4.7 Statistical Methods

All statistical analyses were carried out in R, and pairwise comparisons were made using the Student's *t*‐test (two‐tailed, confidence at 0.95). Model settings were adjusted based on the homoscedasticity of the populations being compared, determined from an *F*‐test of variances. T1 progeny that result from a single T0 plant are excluded from statistical comparison due to lack of biological replicates.

## Author Contributions

M.K.S., L.Y., P.C., T.J., M.C.A. designed the research. L.Y., K.D. and M.K.S. analysed the data. P.C., L.Y., K.D., M.K.S. performed the experiments. M.K.S., M.C.A., I.D.G. and A.M.G.K. wrote and revised the manuscript.

## Conflicts of Interest

The authors declare the following competing interests: M.K.S., L.Y., P.C., K.D., T.J., M.C.A. are or have been employees of Corteva Agriscience.

## Supporting information


**Figure S1:** Analysis of tetrad development during pollen grain development of *spo11‐1* and *rec8* mutants in sorghum. Bars = 5 μm.
**Figure S2:** Examples of plant phenotypes generated from the two‐step synthetic apomixis approach in Tx623/Tx430 hybrid sorghum. (A) Plant morphology of T0 events. Plant second from right resembles sexual hybrids, and the other phenotypes represented 82% of the T0 events. (B) Panicle morphology of T0 event shown second from right in (A). Bars = 10 cm.
**Figure S3:** Parthenogenesis rate (mean ± SE) of F1 controls and T1 progeny from four independent events (Table 3) of both Tx623/Tx430 (A) and Tx623/Macia (B) synthetic apomictic hybrids.
**Figure S4:** SNP marker analysis of progeny from T2, T3 and T4 generations of transgenic event 143 of two‐step synthetic apomixis in Tx623/Tx430 hybrid sorghum. The Tx430 alleles are marked in blue, Tx623 alleles in red, and heterozygous alleles in yellow, and missing marker calls in white.
**Figure S5:** Phenotypic analysis of progeny from an F1 control, T2 and T3 generations of transgenic event 143 of two‐step synthetic apomixis in Tx623/Tx430 hybrid sorghum. (A) Mean panicle length (±SE). Bars with different letters are significantly different (two‐tailed *t*‐test, *p* > 0.05). (B) Mean days to first pollen shed (±SE). Not significantly (ns) different (single factor ANOVA, *p* = 0.654). (C) Mean 1000‐grain weight (±SE). Generations with different letters are significantly different (two‐tailed t‐test, *p* < 0.05).
**Figure S6:** Representative seed samples of the Tx623/Tx430 F1 hybrid control (A), T3 generation progeny from event 143 apomictic hybrid (B), and seed of the subsequent T4 generation (C), bar = 1 cm.
**Figure S7:** Representative flow cytometry histograms of PI‐stained nuclei isolated from young developing seeds of a Tx623/Tx430 hybrid WT control (A), Tx623/Tx430 apomictic event 143 plants (B) and an overlay of the two (C). The WT control seeds contain diploid sporophytic tissue, a diploid immature embryo and triploid developing endosperm (arrow in A and C). The synthetic apomictic hybrid contains diploid sporophytic tissue, a diploid embryo and hexaploid developing endosperm. *X*‐axis shows log relative fluorescent intensity (PI), and the *Y*‐axis is nuclei number.
**Figure S8:** Diagram of *MiMe* loci gene structures and CRISPR target sites. A shows the gene structure of *Sb‐Spo11‐1* with gRNA target sites (in red) and PAM sequence (in green). B shows the gene structure of *Sb‐Rec8* with gRNA target sites (in red) and PAM sequence (in green). C shows the gene structure of *Sb‐OsdL1* and *Sb*‐*OsdL3* with gRNA target sites (in red) and PAM sequence (in green). Diagram generated using *Geneious version 2025.0 created by* Biomatters. Available from https://www.geneious.com.
**Table S1:** Edited alleles of *spo11‐1*, *rec8*, *osdL1*, *osdL3*, and *osdL1 osdL3* in the Tx430 background. The PAM sequence is indicated in blue, and CRISPR/Cas9 induced insertion/deletion indicated in red.
**Table S2:** Plasmids used for *Agrobacterium* transformation to produce transgenic and CRISPR/Cas9 edited events in sorghum.
**Table S3:** Analyses of cytological parthenogenetic embryo development in emasculated *ASGR*‐*BBML2* transgenic Tx430 plants.
**Table S4:** Characterisation of Tx623/Tx430 and Tx623/Macia apomictic T0 CRISPR/Cas9 editing efficiencies at *OsdL1*, *OsdL3*, *Spo11‐1* and *Rec8* gene target sites.
**Table S5:** Characterisation of Tx623/Tx430 apomictic T0 and T1 generation CRISPR/Cas9 edit alleles at *OsdL1*, *OsdL3*, *Spo11‐1* and *Rec8* gene target sites (note that some T0 plants contained more than two unique alleles, suggesting chimerism within the T0 plant).
**Table S6:** Primers sequences used for amplicon analysis of CRISPR/Cas9 edits.

## Data Availability

Novel biological materials described in this publication may be available to the academic community and other not‐for‐profit institutions solely for non‐commercial research purposes upon acceptance and signing of a material transfer agreement between the author's institution and the requester. In some cases, such materials may originally contain genetic elements described in the manuscript that were obtained from a third party (e.g., *DsRed*, *AmCyan*, and *Cas9*), and the authors may not be able to provide materials including third‐party genetic elements to the requester because of certain third‐party contractual restrictions placed on the author's institution. In such cases, the requester will be required to obtain such materials directly from the third party. The authors and the authors' institution do not grant any express or implied permission(s) to the requester to make, use, sell, offer for sale or import third‐party proprietary materials. Obtaining any such permission(s) will be the sole responsibility of the requester. Corteva Agriscience proprietary germplasm will not be made available except at the discretion of Corteva Agriscience and then only in accordance with all applicable governmental regulations.

## References

[pbi70441-bib-0001] Albertsen, M. C. , M. A. Chamberlin , T. W. Fox , S. J. Lawit , and B. R. Loveland . 2013. “Compositions and Methods for Expression of a Sequence in a Reproductive Tissue of a Plant.” US20130180009A1.

[pbi70441-bib-0002] Anand, A. , S. H. Bass , E. Wu , et al. 2018. “An Improved Ternary Vector System for Agrobacterium‐Mediated Rapid Maize Transformation.” Plant Molecular Biology 97: 187–200. 10.1007/s11103-018-0732-y. 29687284 PMC5945794

[pbi70441-bib-0003] Boutilier, K. , R. Offringa , V. K. Sharma , et al. 2002. “Ectopic Expression of BABY BOOM Triggers a Conversion From Vegetative to Embryonic Growth.” Plant Cell 14: 1737–1749. 10.1105/tpc.001941. 12172019 PMC151462

[pbi70441-bib-0004] Che, P. , A. Anand , E. Wu , et al. 2018. “Developing a Flexible, High‐Efficiency Agrobacterium‐Mediated Sorghum Transformation System With Broad Application.” Plant Biotechnology Journal 16: 1388–1395. 10.1111/pbi.12879. 29327444 PMC5999184

[pbi70441-bib-0005] Che, P. , E. Wu , M. K. Simon , et al. 2022. “Wuschel2 Enables Highly Efficient CRISPR/Cas‐Targeted Genome Editing During Rapid de Novo Shoot Regeneration in Sorghum.” Communications Biology 5: 344. 10.1038/s42003-022-03308-w. 35410430 PMC9001672

[pbi70441-bib-0006] Conner, J. A. , M. Mookkan , H. Huo , K. Chae , and P. Ozias‐Akins . 2015. “A Parthenogenesis Gene of Apomict Origin Elicits Embryo Formation From Unfertilized Eggs in a Sexual Plant.” Proceedings of the National Academy of Sciences of the United States of America 112: 11205–11210. 10.1073/pnas.1505856112. 26305939 PMC4568661

[pbi70441-bib-0007] Conner, J. A. , M. Podio , and P. Ozias‐Akins . 2017. “Haploid Embryo Production in Rice and Maize Induced by PsASGR‐BBML Transgenes.” Plant Reproduction 30: 41–52. 10.1007/s00497-017-0298-x. 28238020

[pbi70441-bib-0008] Dan, J. , Y. Xia , Y. Wang , et al. 2024. “One‐Line Hybrid Rice With High‐Efficiency Synthetic Apomixis and Near‐Normal Fertility.” Plant Cell Reports 43: 79. 10.1007/s00299-024-03154-6. 38400858 PMC10894110

[pbi70441-bib-0009] de Wet, J. M. J. 1978. “Special Paper: Systematics and Evoluation of Sorghum Sect. Sorghum (Graminaea).” American Journal of Botany 65: 477–484. 10.1002/j.1537-2197.1978.tb06096.x.

[pbi70441-bib-0010] d'Erfurth, I. , S. Jolivet , N. Froger , O. Catrice , M. Novatchkova , and R. Mercier . 2009. “Turning Meiosis Into Mitosis.” PLoS Biology 7: e1000124. 10.1371/journal.pbio.1000124. 19513101 PMC2685454

[pbi70441-bib-0011] Doležel, J. , and J. Bartos . 2005. “Plant DNA Flow Cytometry and Estimation of Nuclear Genome Size.” Annals of Botany 95: 99–110. 10.1093/aob/mci005. 15596459 PMC4246710

[pbi70441-bib-0012] Doležel, J. , P. Binarová , and S. Lcretti . 1989. “Analysis of Nuclear DNA Content in Plant Cells by Flow Cytometry.” Biologia Plantarum 31: 113–120. 10.1007/BF02907241.

[pbi70441-bib-0013] Fomicheva, M. , Y. Kulakov , K. Alyokhina , and E. Domblides . 2024. “Spontaneous and Chemically Induced Genome Doubling and Polyploidization in Vegetable Crops.” Horticulturae 10, no. 6: 551. 10.3390/horticulturae10060551.

[pbi70441-bib-0014] Gilles, L. M. , A. Khaled , J. B. Laffaire , et al. 2017. “Loss of Pollen‐Specific Phospholipase NOT LIKE DAD Triggers Gynogenesis in Maize.” EMBO Journal 36: 707–717. 10.15252/embj.201796603. 28228439 PMC5350562

[pbi70441-bib-0015] Hand, M. L. , and A. M. G. Koltunow . 2014. “The Genetic Control of Apomixis: Asexual Seed Formation.” Genetics 197: 441–450. 10.1534/genetics.114.163105. 24939990 PMC4063905

[pbi70441-bib-0016] Hanna, W. W. , and E. C. Bashaw . 1987. “Apomixis: Its Identification and Use in Plant Breeding.” Crop Science 27: 1136–1139. 10.2135/cropsci1987.0011183X002700060010x.

[pbi70441-bib-0017] Heidemann, B. , E. Primetis , I. E. Zahn , and C. J. Underwood . 2025. “To Infinity and Beyond: Recent Progress, Bottlenecks, and Potential of Clonal Seeds by Apomixis.” Plant Journal 121: e70054. 10.1111/tpj.70054. PMC11843595 39981717

[pbi70441-bib-0018] Hossain, S. , N. Islam , M. Rahman , M. G. Mostofa , and A. R. Khan . 2022. “Sorghum: A Prospective Crop for Climatic Vulnerability, Food and Nutritional Security.” Journal of Agriculture and Food Research 8: 100300. 10.1016/j.jafr.2022.100300.

[pbi70441-bib-0019] Huang, Y. , X. Meng , Y. Rao , et al. 2025. “OsWUS‐Driven Synthetic Apomixis in Hybrid Rice.” Plant Communications 6: 101136. 10.1016/j.xplc.2024.101136. 39305015 PMC11783873

[pbi70441-bib-0020] Iwata, E. , S. Ikeda , N. Abe , et al. 2012. “Roles of GIG1 and UVI4 in Genome Duplication in *Arabidopsis thaliana* .” Plant Signaling & Behavior 7, no. 9: 1079–1081. 10.4161/psb.21133. 22899078 PMC3489631

[pbi70441-bib-0021] Iwata, E. , S. Ikeda , S. Matsunaga , et al. 2011. “GIGAS CELL1, a Novel Negative Regulator of the Anaphase‐Promoting Complex/Cyclosome, Is Required for Proper Mitotic Progression and CELL Fate Determination in Arabidopsis.” Plant Cell 23, no. 12: 4382–4393. 10.1105/tpc.111.092049. 22167058 PMC3269872

[pbi70441-bib-0022] Kazungu, F. K. , E. M. Muindi , and J. M. Mulinge . 2023. “Overview of Sorghum (*Sorghum bicolor*. L), Its Economic Importance, Ecological Requirements and Production Constraints in Kenya.” International Journal of Plant and Soil Science 35, no. 1: 62–71. 10.9734/ijpss/2023/v35i12744.

[pbi70441-bib-0023] Kelliher, T. , D. Starr , L. Richbourg , et al. 2017. “MATRILINEAL, a Sperm‐Specific Phospholipase, Triggers Maize Haploid Induction.” Nature 542: 105–109. 10.1038/nature20827. 28114299

[pbi70441-bib-0024] Khalifa, M. , and E. A. B. Eltahir . 2023. “Assessment of Global Sorghum Production, Tolerance, and Climate Risk.” Frontiers in Sustainable Food Systems 7: 1184373. 10.3389/fsufs.2023.1184373.

[pbi70441-bib-0025] Khanday, I. , D. Skinner , B. Yang , R. Mercier , and V. Sundaresan . 2019. “A Male‐Expressed Rice Embryogenic Trigger Redirected for Asexual Propagation Through Seeds.” Nature 565: 91–95. 10.1038/s41586-018-0785-8. 30542157

[pbi70441-bib-0026] Koltunow, A. M. , and U. Grossniklaus . 2003. “Apomixis: A Developmental Perspective.” Annual Review of Plant Biology 54: 547–574. 10.1146/annurev.arplant.54.110901.160842. 14503003

[pbi70441-bib-0027] Leblanc, O. , D. Grimanelli , N. Islam‐Faridi , J. Berthaud , and Y. Savidan . 1996. “Reproductive Behavior in Maize‐Tripsacum Polyhaploid Plants: Implications for the Transfer of Apomixis Into Maize.” Journal of Heredity 87: 108–111. 10.1093/oxfordjournals.jhered.a022964.

[pbi70441-bib-0028] Liu, C. , Z. He , Y. Zhang , et al. 2023. “Synthetic Apomixis Enables Stable Transgenerational Transmission of Heterotic Phenotypes in Hybrid Rice.” Plant Communications 4: 100470. 10.1016/j.xplc.2022.100470. 36325606 PMC10030361

[pbi70441-bib-0029] Liu, C. , X. Li , D. Meng , et al. 2017. “A 4‐Bp Insertion at ZmPLA1 Encoding a Putative Phospholipase A Generates Haploid Induction in Maize.” Molecular Plant 10: 520–522. 10.1016/j.molp.2017.01.011. 28179149

[pbi70441-bib-0030] Marimuthu, M. P. A. , S. Jolivet , M. Ravi , et al. 2011. “Synthetic Clonal Reproduction Through Seeds.” Science 331: 876. 10.1126/science.1199682. 21330535

[pbi70441-bib-0031] Massel, K. , Y. Lam , J. Hintzsche , N. Lester , J. R. Botella , and I. D. Godwin . 2022. “Endogenous U6 Promoters Improve CRISPR/Cas9 Editing Efficiencies in *Sorghum bicolor* and Show Potential for Applications in Other Cereals.” Plant Cell Reports 41: 489–492. 10.1007/s00299-021-02816-z. 34854968

[pbi70441-bib-0032] Mieulet, D. , S. Jolivet , M. Rivard , et al. 2016. “Turning Rice Meiosis Into Mitosis.” Cell Research 26: 1242–1254. 10.1038/cr.2016.117. 27767093 PMC5099866

[pbi70441-bib-0033] Nogler, G. A. 1984. “Gametophytic Apomixis.” In Embryology of Angiosperms, edited by B. M. Johri , 475–518. Springer Berlin Heidelberg. 10.1007/978-3-642-69302-1_10.

[pbi70441-bib-0034] Ravi, M. , and S. W. Chan . 2010. “Haploid Plants Produced by Centromere‐Mediated Genome Elimination.” Nature 464: 615–618. 10.1038/nature08842. 20336146

[pbi70441-bib-0035] Ren, H. , K. Shankle , M. J. Cho , M. Tjahjadi , I. Khanday , and V. Sundaresan . 2024. “Synergistic Induction of Fertilization‐Independent Embryogenesis in Rice Egg Cells by Paternal‐Genome‐Expressed Transcription Factors.” Nature Plants 10: 1892–1899. 10.1038/s41477-024-01848-z. 39533074

[pbi70441-bib-0036] Richards, A. J. 2003. “Apomixis in Flowering Plants: An Overview.” Philosophical Transactions of the Royal Society of London. Series B, Biological Sciences 358: 1085–1093. 10.1098/rstb.2003.1294. 12831474 PMC1693200

[pbi70441-bib-0037] Ross, K. J. , P. Fransz , and G. H. Jones . 1996. “A Light Microscopic Atlas of Meiosis in *Arabidopsis thaliana* .” Chromosome Research 4, no. 7: 507–516. 10.1007/BF02261778. 8939362

[pbi70441-bib-0038] Song, M. , F. Li , Z. Chen , et al. 2024. “Engineering High‐Frequency Apomixis With Normal Seed Production in Hybrid Rice.” IScience 27: 111479. 10.1016/j.isci.2024.111479. 39720515 PMC11667186

[pbi70441-bib-0039] Song, M. , W. Wang , C. Ji , et al. 2024. “Simultaneous Production of High‐Frequency Synthetic Apomixis With High Fertility and Improved Agronomic Traits in Hybrid Rice.” Molecular Plant 17, no. 1: 4–7. 10.1016/j.molp.2023.11.007. 37990497

[pbi70441-bib-0040] Underwood, C. J. , and R. Mercier . 2022. “Engineering Apomixis: Clonal Seeds Approaching the Fields.” Annual Review of Plant Biology 73: 201–225. 10.1146/annurev-arplant-102720-013958. 35138881

[pbi70441-bib-0041] Underwood, C. J. , K. Vijverberg , D. Rigola , et al. 2022. “A PARTHENOGENESIS Allele From Apomictic Dandelion Can Induce Egg Cell Division Without Fertilization in Lettuce.” Nature Genetics 54: 84–93. 10.1038/s41588-021-00984-y. 34992267

[pbi70441-bib-0042] Vernet, A. , D. Meynard , Q. Lian , et al. 2022. “High‐Frequency Synthetic Apomixis in Hybrid Rice.” Nature Communications 13: 7963. 10.1038/s41467-022-35679-3. PMC9794695 36575169

[pbi70441-bib-0043] Wang, C. , Q. Liu , Y. Shen , et al. 2019. “Clonal Seeds From Hybrid Rice by Simultaneous Genome Engineering of Meiosis and Fertilization Genes.” Nature Biotechnology 37: 283–286. 10.1038/s41587-018-0003-0. 30610223

[pbi70441-bib-0044] Wang, Y. , R. R. Fuentes , W. M. van Rengs , et al. 2024. “Harnessing Clonal Gametes in Hybrid Crops to Engineer Polyploid Genomes.” Nature Genetics 56: 1075–1079. 10.1038/s41588-024-01750-6. 38741016 PMC11176054

[pbi70441-bib-0045] Wei, X. , C. Liu , X. Chen , et al. 2023. “Synthetic Apomixis With Normal Hybrid Rice Seed Production.” Molecular Plant 16: 489–492. 10.1016/j.molp.2023.01.005. 36609144

[pbi70441-bib-0046] Wu, E. , B. Lenderts , K. Glassman , et al. 2014. “Optimized Agrobacterium‐Mediated Sorghum Transformation Protocol and Molecular Data of Transgenic Sorghum Plants.” In Vitro Cellular & Developmental Biology. Plant 50: 9–18. 10.1007/s11627-013-9583-z. 26316679 PMC4544465

[pbi70441-bib-0047] Xie, E. , Y. Li , D. Tang , Y. Lv , Y. Shen , and Z. Cheng . 2019. “A Strategy for Generating Rice Apomixis by Gene Editing.” Journal of Integrative Plant Biology 61: 911–916. 10.1111/jipb.12785. 30697955

[pbi70441-bib-0048] Ye, H. , M. Louden , and J. A. T. Reinders . 2024. “A Novel In Vivo Genome Editing Doubled Haploid System for *Zea mays* L.” Nature Plants 10: 1493–1501. 10.1038/s41477-024-01795-9. 39333351

[pbi70441-bib-0049] Zhi, L. , S. TeRonde , S. Meyer , et al. 2015. “Effect of Agrobacterium Strain and Plasmid Copy Number on Transformation Frequency, Event Quality and Usable Event Quality in an Elite Maize Cultivar.” Plant Cell Reports 34: 745–754. 10.1007/s00299-014-1734-0. 25558819

