# Supplementary material for: Induction of Synthetic Apomixis in Two Sorghum Hybrids Enables Seed Yield and Genotype Preservation Over Multiple Generations
Source: Plant Biotechnol J. 2025 Nov 5;24(3):1712–24. doi: 10.1111/pbi.70441 (PMC12946465; doi:10.1111/pbi.70441)
Supplement: Supplementary file 1 — Figure S1: Analysis of tetrad development during pollen grain development of spo11‐1 and rec8 mutants in sorghum. Bars = 5 μm. Figure S2: Examples of plant phenotypes generated from the two‐step synthetic apomixis approach in Tx623/Tx430 hybrid sorghum. (A) Plant morphology of T0 events. Plant second from right resembles sexual hybrids, and the other phenotypes represented 82% of the T0 events. (B) Panicle morphology of T0 event shown second from right in (A). Bars = 10 cm. Figure S3: Parthenogenesis rate (mean ± SE) of F1 controls and T1 progeny from four independent events (Table 3) of both Tx623/Tx430 (A) and Tx623/Macia (B) synthetic apomictic hybrids. Figure S4: SNP marker analysis of progeny from T2, T3 and T4 generations of transgenic event 143 of two‐step synthetic apomixis in Tx623/Tx430 hybrid sorghum. The Tx430 alleles are marked in blue, Tx623 alleles in red, and heterozygous alleles in yellow, and missing marker calls in white. Figure S5: Phenotypic analysis of progeny from an F1 control, T2 and T3 generations of transgenic event 143 of two‐step synthetic apomixis in Tx623/Tx430 hybrid sorghum. (A) Mean panicle length (±SE). Bars with different letters are significantly different (two‐tailed t‐test, p > 0.05). (B) Mean days to first pollen shed (±SE). Not significantly (ns) different (single factor ANOVA, p = 0.654). (C) Mean 1000‐grain weight (±SE). Generations with different letters are significantly different (two‐tailed t‐test, p < 0.05). Figure S6: Representative seed samples of the Tx623/Tx430 F1 hybrid control (A), T3 generation progeny from event 143 apomictic hybrid (B), and seed of the subsequent T4 generation (C), bar = 1 cm. Figure S7: Representative flow cytometry histograms of PI‐stained nuclei isolated from young developing seeds of a Tx623/Tx430 hybrid WT control (A), Tx623/Tx430 apomictic event 143 plants (B) and an overlay of the two (C). The WT control seeds contain diploid sporophytic tissue, a diploid immature embryo and triploid de [file PBI-24-1712-s001.docx]

**Supplemental Information**


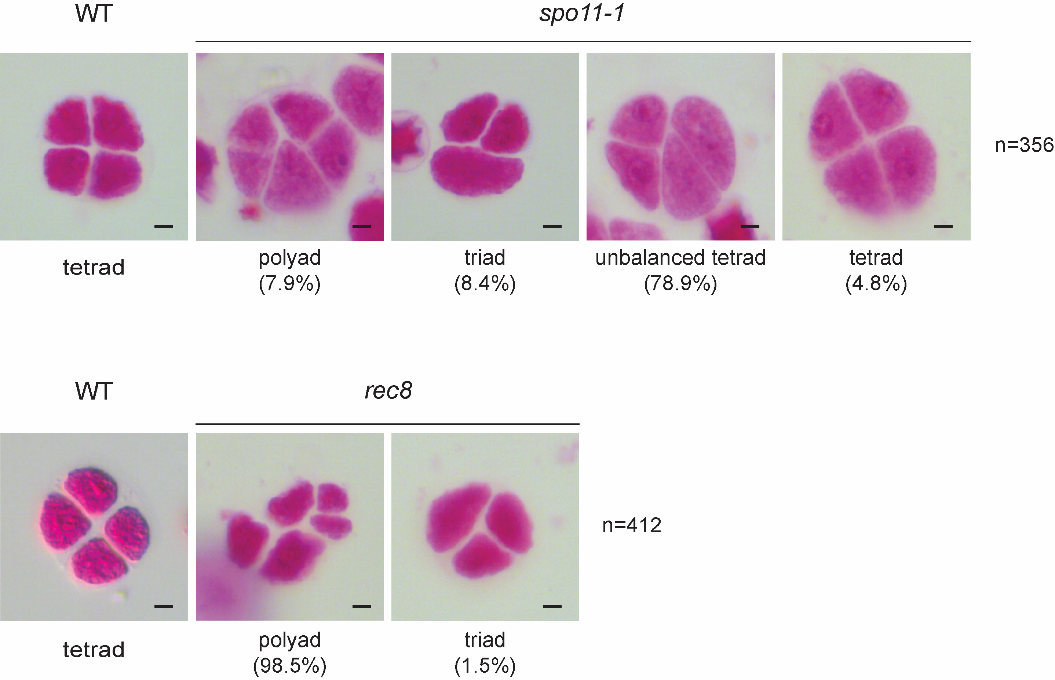


Supplemental Figure 1. Analysis of tetrad development during pollen grain development of *spo11-1* and *rec8* mutants in sorghum. Bars = 5 µm.


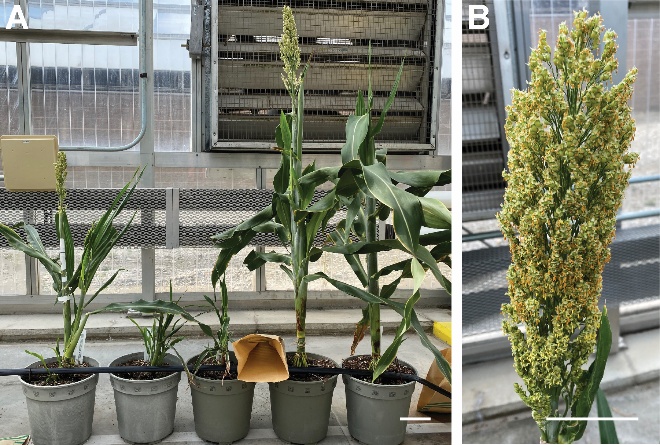


Supplemental Figure 2. Examples of plant phenotypes generated from the two-step synthetic apomixis approach in Tx623/Tx430 hybrid sorghum. (A) Plant morphology of T0 events. Plant second from right resembles sexual hybrids, and the other phenotypes represented 82% of the T0 events. (B) Panicle morphology of T0 event shown second from right in (A). Bars = 10 cm.


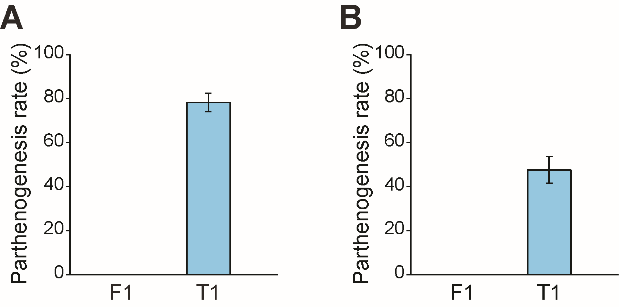


Supplemental Figure 3. Parthenogenesis rate (mean ± se) of F1 controls and T1 progeny from four independent events (Table 3) of both Tx623/Tx430 (A) and Tx623/Macia (B) synthetic apomictic hybrids.


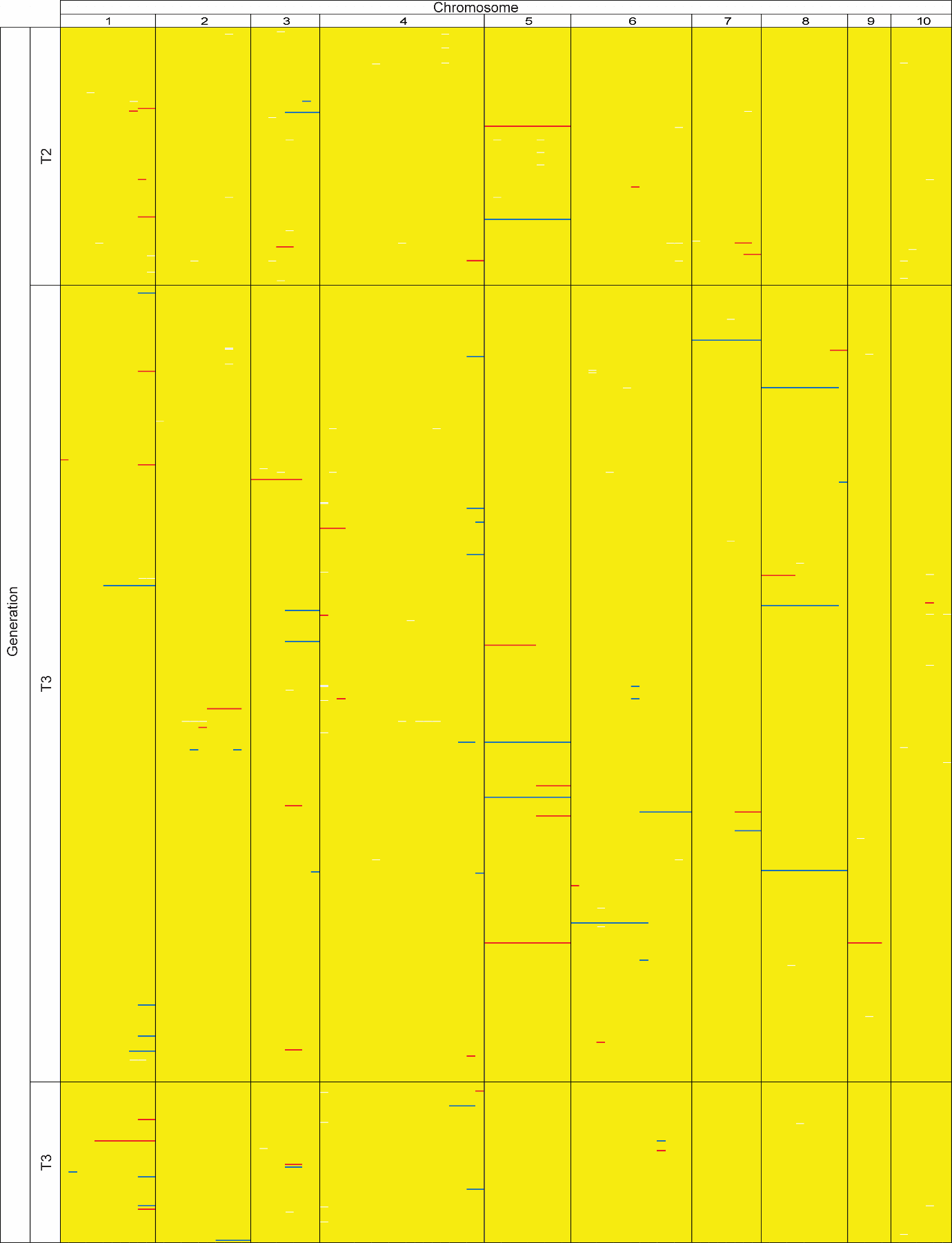


Supplemental Figure 4. SNP marker analysis of progeny from T2, T3 and T4 generations of transgenic event 143 of two-step synthetic apomixis in Tx623/Tx430 hybrid sorghum.  The Tx430 alleles are marked in blue, Tx623 alleles in red, and heterozygous alleles in yellow, and missing marker calls in white.


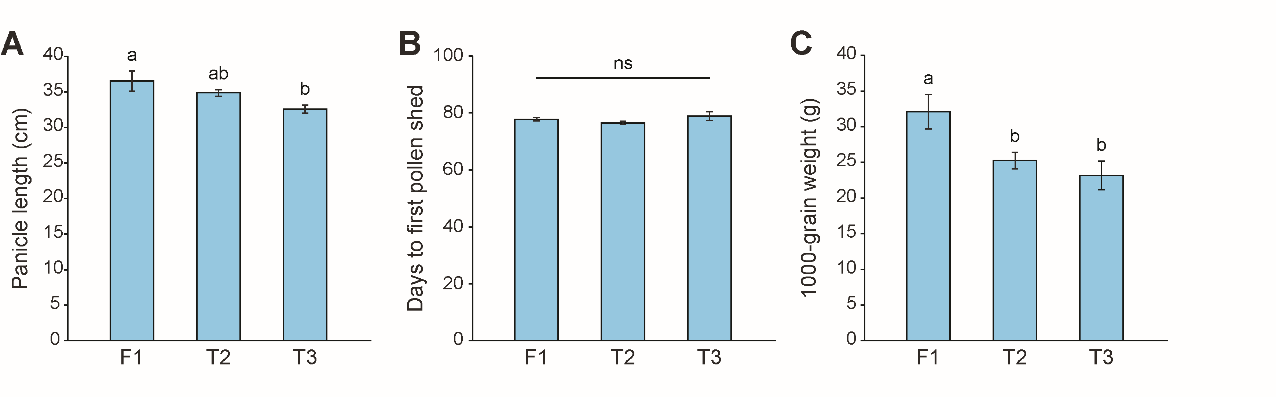


Supplemental Figure 5. Phenotypic analysis of progeny from an F1 control, T2 and T3 generations of transgenic event 143 of two-step synthetic apomixis in Tx623/Tx430 hybrid sorghum. (A) Mean panicle length (± se). Bars with different letters are significantly different (two-tailed t-test, p > 0.05). (B) Mean days to first pollen shed (± se). Not significantly (ns) different (single factor ANOVA, P=0.654). (C) Mean 1000-grain weight (± se). Generations with different letters are significantly different (two-tailed t-test, p < 0.05).


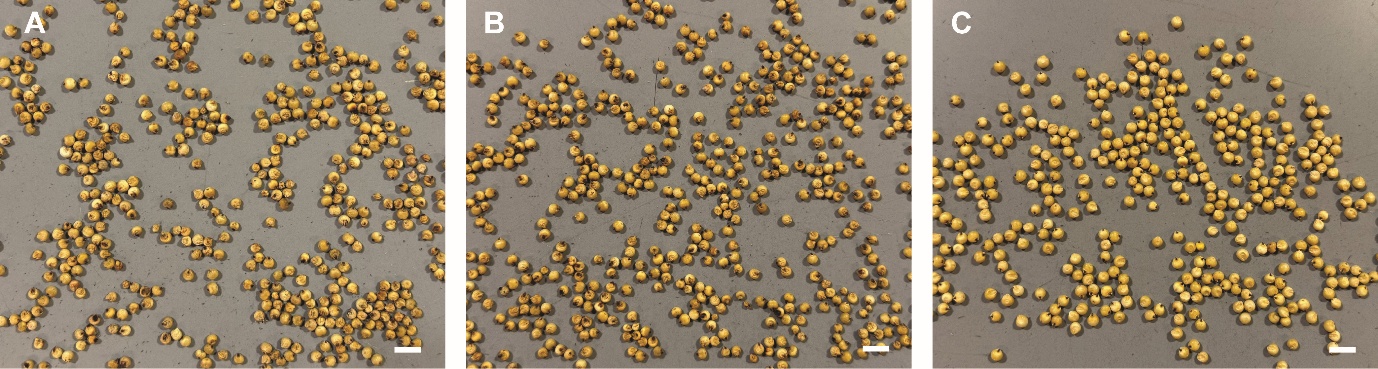


Supplemental Figure 6. Representative seed samples of the Tx623/Tx430 F1 hybrid control (A), T3 generation progeny from event 143 apomictic hybrid (B), and seed of the subsequent T4 generation (C), bar = 1cm.


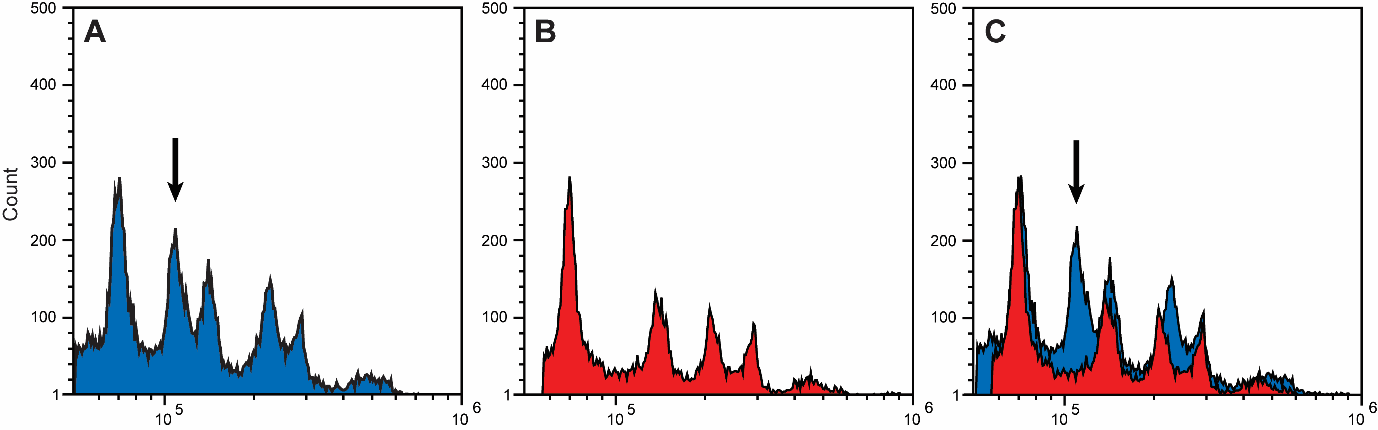


Supplemental Figure 7. Representative flow cytometry histograms of PI-stained nuclei isolated from young developing seeds of a Tx623/Tx430 hybrid WT control (A), Tx623/Tx430 apomictic event 143 plants (B) and an overlay of the two (C). The WT control seeds contain diploid sporophytic tissue, a diploid immature embryo and triploid developing endosperm (arrow in A and C). The synthetic apomictic hybrid contains diploid sporophytic tissue, a diploid embryo and hexaploid developing endosperm. X axis shows log relative fluorescent intensity (PI), and the Y axis is nuclei number.


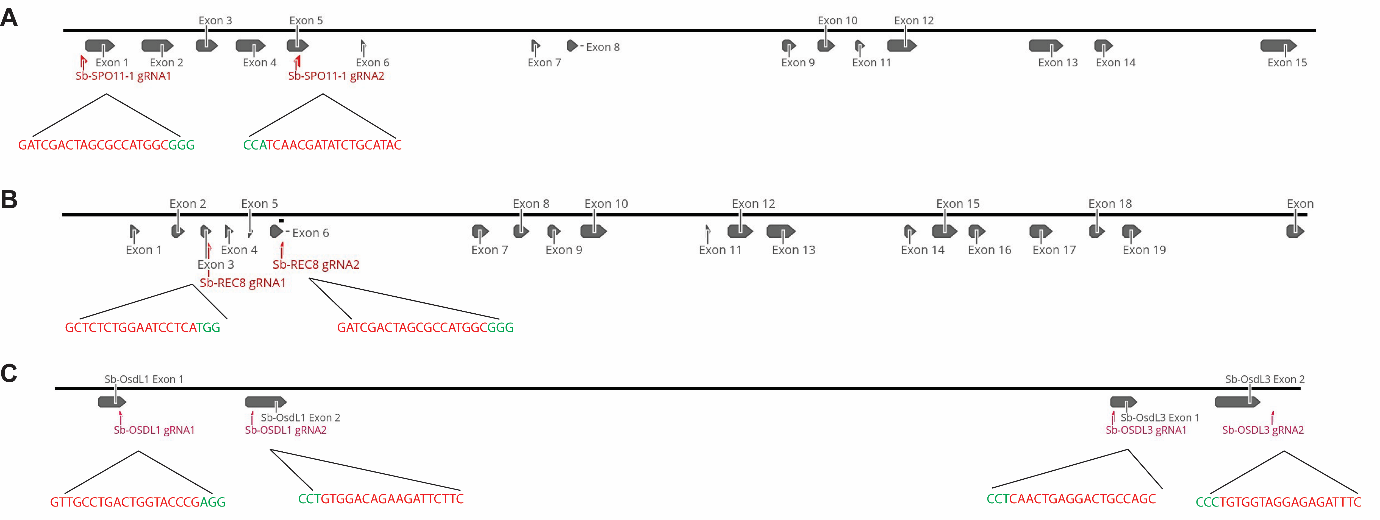
 Supplemental Figure 8.  Diagram of *MiMe* loci gene structures and CRISPR target sites.  A shows the gene structure of *Sb-Spo11-1* with gRNA target sites (in red) and PAM sequence (in green). B shows the gene structure of *Sb-Rec8* with gRNA target sites (in red) and PAM sequence (in green) . C shows the gene structure of *Sb-OsdL1* and *Sb*-*OsdL3* with gRNA target sites (in red) and PAM sequence (in green). Diagram generated using *Geneious version 2025.0 created by Biomatters. Available from* [*https://www.geneious.com*](https://www.geneious.com/)

Supplemental Table 1. Edited alleles of *spo11-1*, *rec8*, *osdL1*, *osdL3*, and *osdL1* *osdL3* in the Tx430 background. The PAM sequence is indicated in blue, and CRISPR/Cas9 induced insertion/deletion indicated in red.

| Gene | Mutant | gRNA1 mutation | gRNA1 mutation | gRNA2 mutation | gRNA2 mutation | Genetic background |
| --- | --- | --- | --- | --- | --- | --- |
| *Spo11-1* | WT | GATCGACTAGCGCCATGGCGGG | n/a | CCATCAACGATATCTGCATAC | n/a | Tx430 |
|  | Mutant 1 | GATCGACTAGCGCC--GGCGGG | frameshift | CCATCAAACGATATCTGCATAC | frameshift | Tx430 |
|  | Mutant 2 | GATCGACTAGCGC--TGGCGGG | frameshift | CCATCAAACGATATCTGCATAC | frameshift | Tx430 |
|  | Mutant 3 | GATCGACTAGCG--TGGCGGG/  GATCGACTAGCGC---GGCGGG | frameshift | CCATCAAACGATATCTGCATAC/  CCATCACACGATATCTGCATAC | frameshift | Tx430 |
|  | Mutant 4 | GATCGACTAGCGC--TGGCGGG/  GATCGACTAGCGCC--GGCGGG | frameshift | CCATC-ACGATATCTGCATAC/  C---CAACGATATCTGCATAC | frameshift/  in-frame | Tx430 |
| *Rec8* | WT | GCTCTCTGGAATCCTCATGG | n/a | GCTTGGGCTTTACCCTTGGG | n/a | Tx430 |
|  | Mutant 1 | No editing | n/a | GCTTGGG------------- (-13) | frameshift | Tx430 |
|  | Mutant 2 | No editing | n/a | GCTTGGGCTTT--CTTGGG/  GCTTGGGCTTTACCACTTGGG | frameshift | Tx430 |
|  | Mutant 3 | No editing | n/a | GCTTGGGCTTTAC-CTTGGG/  GCTTGGGCTTTACCTCTTGGG | frameshift | Tx430 |
|  | Mutant 4 | No editing | n/a | GCTTGGGCTTTAC-CTTGGG/  GCTTGGG------------- (-13) | frameshift | Tx430 |
| *OsdL1* | WT | GTTGCCTGACTGGTACCCGAGG | n/a | CCTGTGGACAGAAGATTCTTC | n/a | Tx430 |
|  | Mutant 1 | GTTGCCTGACTGGTACT----- (-1233)/  GTTGCCTGACTGGTACACCGAGG | frameshift | (-1233) ------GACAGAAGATTCTTC/  CCTGTGAGACAGAAGATTCTTC | frameshift | Tx430 |
|  | Mutant 2 | GTTGCCTGACTGGTACACCGAGG | frameshift | No editing | n/a | Tx430 |
| *OsdL3* | WT | CTTCAACTGAGGACTGCCAGC | n/a | CCCTGTGGTAGGAGAGATTTC | n/a | Tx430 |
|  | Mutant 1 | (-1537) ----------------------/  CTTCAA-TGAGGACTGCCAGC | frameshift | ( -1537) ---------AGGAGAGATTTC/  CCCTGTGGGTAGGAGAGATTTC | frameshift | Tx430 |
|  | Mutant 2 | CTTCAATCTGAGGACTGCCAGC/  CTTCAAACTGAGGACTGCCAGC | frameshift | CCCTGTGGGTAGGAGAGATTTC | frameshift | Tx430 |
| *OsdL1/OsdL3* | WT | GTTGCCTGACTGGTACCCGAGG | n/a | CCTCAACTGAGGACTGCCAGC | n/a | Tx430 |
|  | Mutant 1 | GTTG------ (-12) ------CCGAGG /  GTTGCCTGACTGGTACTCCGAGG | in-frame/ frameshift | CCTCAA-TGAGGACTGCCAGC | frameshift | Tx430 |
|  | Mutant 2 | GTTGCCTGACTGGTA-CCGAGG/  GTTGCCTGACTGGTACTCCGAGG | frameshift | CCTCAAAACTGAGGACTGCCAGC  CCTCAA--GAGGACTGCCAGC | frameshift | Tx430 |
|  | Mutant 3 | GTTGCC---------------- (-30) | in-frame | (-11) -------TGAGGACTGCCAGC/  CCTCAA---- (-9) -----GCCAGC | frameshift/  in-frame | Tx430 |
|  | Mutant 4 | GTTG----- (-11) ------CCCGAGG | frameshift | CCTCAA-TGAGGACTGCCAGC/  CCTCAACTCCTGAGGACTGCCAGC | frameshift/  in-frame | Tx430 |
|  | Mutant 5 | GTTGCCTGACTGGTACCCCGAGG/  GTTG------- (-15) --------AGG | frameshift/  in-frame | CCTCAA-TGAGGACTGCCAGC/  (-23) --------------------C | frameshift | Tx430 |
|  | Mutant 6 | GTTGCCTGACTGGTACCCCGAGG | frameshift | CCTCAA-TGAGGACTGCCAGC | frameshift | Tx430 |

Supplemental Table 2. Plasmids used for *Agrobacterium* transformation to produce transgenic and CRISPR/Cas9 edited events in sorghum.

| Plasmid name | Promoter | Gene | Selectable marker | Transformation method | Genetic background |
| --- | --- | --- | --- | --- | --- |
| PHP83000 | *Cenchrus* *CS-ASGR-BBML2* | *ASGR-BBML2* gDNA | *PMI* | Conventional | Tx430 |
| PHP82772 | *Cenchrus* *CS-ASGR-BBML2* | *ASGR-BBML2* cDNA | *PMI* | Conventional | Tx430 |
| PHP86483 | Maize *ZM-DD45* | *ASGR-BBML2* gDNA | *HRA* | morphogenic gene-mediated | Tx430 |
| PHP86482 | Maize *ZM-DD45* | *ASGR-BBML2* cDNA | *HRA* | morphogenic gene-mediated | Tx430 |
| PHP86484 | Maize *ZM-RKD2* | *ASGR-BBML2* gDNA | *HRA* | morphogenic gene-mediated | Tx430 |
| PHP86796 | Maize *ZM-RKD2* | *ASGR-BBML2* cDNA | *HRA* | morphogenic gene-mediated | Tx430 |
| PHP94292 | Maize *ZM-DD45* | *ASGR-BBML2* cDNA | *NPTII* (excised) | Wus2/CRE-mediated marker-free | Tx430 |

| Plasmid name | Gene targeted | Gene ID | gRNA number | gRNA sequence | gRNA sequence | Transformation method | Genetic background |
| --- | --- | --- | --- | --- | --- | --- | --- |
| PHP85484 | *Spo11-1* | Sobic.001G087000 | 2 | GATCGACTAGCGCCATGGC | GTATGCAGATATCGTTGA | Conventional | Tx430 |
| PHP83595 | *Rec8* | Sobic.009G245400 | 2 | GCTCTCTGGAATCCTCA | GCTTGGGCTTTACCCTT | Conventional | Tx430 |
| PHP83596 | *OsdL1* | Sobic.004G198100 | 2 | GTTGCCTGACTGGTACCCG | GAAGAATCTTCTGTCCAC | Conventional | Tx430 |
| PHP83436 | *OsdL3* | Sobic.004G198200 | 2 | GCTGGCAGTCCTCAGTTG | GAAATCTCTCCTACCACA | Conventional | Tx430 |
| PHP85930 | *OsdL1 and OsdL3* | See above | 2 | GTTGCCTGACTGGTACCCG | GCTGGCAGTCCTCAGTTG | Conventional | Tx430 |
| PHP110251 | *OsdL1 and OsdL3* | See above | 2 | GTTGCCTGACTGGTACCCG | GCTGGCAGTCCTCAGTTG | morphogenic gene-mediated | Tx430 |
| PHP101035 | *MiMe* | See above | 4 | GATCGACTAGCGCCATGGC | GCTTGGGCTTTACCCTT | Altruistic | Tx623/Tx430 |
|  |  |  |  | GTTGCCTGACTGGTACCCG | GCTGGCAGTCCTCAGTTG |  |  |
| PHP105181 | *MiMe* | See above | 4 | GATCGACTAGCGCCATGGC | GCTTGGGCTTTACCCTT | Altruistic | Tx623/Macia |
|  |  |  |  | GTTGCCTGACTGGTACCCG | GCTGGCAGTCCTCAGTTG |  |  |

Supplemental Table 3. Analyses of cytological parthenogenetic embryo development in emasculated *ASGR-BBML2* transgenic Tx430 plants

| **Construct** | **Events evaluated** | **Total ovules evaluated** | **Average parthenogenetic embryo development (se)** | **Range of parthenogenetic embryo development** |
| --- | --- | --- | --- | --- |
|  |  |  |  |  |
| ASGR-BBML2 pro:  ASGR-BBML2 gDNA | 11 | 255 | 30.84% (5.34) | 0% - 53.33% |
| ASGR-BBML2 pro:  ASGR-BBML2 cDNA | 8 | 152 | 14.80% (5.16) | 0% - 44% |
| ZM-DD45 pro:  ASGR-BBML2 gDNA | 6 | 136 | 45.25% (2.14) | 40% - 51.85% |
| ZM-DD45 pro:  ASGR-BBML2 cDNA | 8 | 185 | 41.72% (4.31) | 18.18% - 54.17% |
| ZM-RKD2 pro:  ASGR-BBML2 gDNA | 6 | 133 | 47.02% (4.04) | 34.78% - 62.5% |
| ZM-RKD2 pro:  ASGR-BBML2 cDNA | 5 | 133 | 19.38% (5.04) | 0% - 28.57% |

Supplemental Table 4. Characterization of Tx623/Tx430 and Tx623/Macia apomictic T0 CRISPR/Cas9 editing efficiencies at *OsdL1*, *OsdL3*, *Spo11-1* and *Rec8* gene target sites.

| **Hybrid** | **T0 plants analyzed** | **Editing target site** | **Editing frequency** | **Biallelic frameshift allele editing frequency** | **Combined biallelic frameshift allele editing frequency** |
| --- | --- | --- | --- | --- | --- |
| Tx623/Tx430 | 150 | *OsdL1*-CR3 | 45.3% | 24.7% | 13.3% |
|  |  | *OsdL3*-CR1 | 46.7% | 30.7% |  |
|  |  | *Rec8*-CR4 | 45.3% | 42% |  |
|  |  | *Spo11-1*-CR1 | 47.3% | 39.3% |  |
| Tx623/Macia | 160 | *OsdL1*-CR3 | 51.3% | 25.6% | 11.9% |
|  |  | *OsdL3*-CR1 | 53.1% | 28.1% |  |
|  |  | *Rec8*-CR4 | 51.9% | 45.6% |  |
|  |  | *Spo11-1*-CR1 | 51.9% | 48.8% |  |

Supplemental Table 5. Characterization of Tx623/Tx430 apomictic T0 and T1 generation CRISPR/Cas9 edit alleles at *OsdL1*, *OsdL3*, *Spo11-1* and *Rec8* gene target sites. (note that some T0 plants contained more than two unique alleles, suggesting chimerism within the T0 plant).

* many unique alleles identified, each individual plant contained a single allele

| SRH type | Event | *Spo11-1* | *Rec8* | *OsdL1* | *OsdL3* |
| --- | --- | --- | --- | --- | --- |
| Tx623/Tx430 SRH | 4 | -19bp | +1bp/-1bp | -11bp | -1bp |
|  | 13 | -2bp | +1bp/-1bp | +1bp/-1bp | -16bp/-23bp |
|  | 21 | -2bp/-3bp | -1bp | +2bp | -16bp |
|  | 31 | -2bp | +1bp/-13bp | +1bp/-1bp/-7bp/-11bp | -2bp/-23bp/+1bp |
|  | 40 | +1bp/-2bp | +1bp/+1bp | +1bp/+1bp/-1bp | +1bp/-1bp |
|  | 52 | -2bp/-4bp | +1bp/+1bp | +1bp | -1bp |
|  | 66 | +1bp/-2bp | -1bp | +1bp | +1bp/+2bp/-1bp/-16bp |
|  | 77 | +1bp/-2bp | +1bp/+1bp | +1bp/-1bp | +1bp/-23bp |
|  | 79 | -3bp/-55bp | +1bp/-13bp | -2bp | -1bp/-16bp |
|  | 86 | +1bp/-3bp | +1bp/-1bp | +1bp/-11bp | -16bp/-18bp |
|  | 87 | -2bp | +1bp/-7bp | +1bp | -16bp |
|  | 91 | -2bp/-2bp | -1bp/+3bp | +2bp/+12bp | +1bp/+1bp/+2bp |
|  | 93 | +1bp/-1bp | +1bp/+1bp | -6bp | +2bp/+9bp |
|  | 98 | -1bp/-3bp | +1bp/+1bp | +1bp/-38bp | +2bp/+3bp |
|  | 100 | SNP/-2bp | +1bp | -1bp/-6bp | +1bp/-41bp |
|  | 104 | SNP/-2bp | +1bp/+1bp | +1bp/-11bp | -1bp |
|  | 109 | -3bp/-8bp | +1bp | -6bp/-4bp/-15bp | +1bp/+2bp/-40bp |
|  | 111 | -2bp/-6bp | +1bp/+1bp | -5bp/-31bp | (-3+3,+SNP)/-1bp |
|  | 118 | +1bp/-9bp | +1bp/-1bp | +1bp/-1bp | +1bp/+2bp/+2bp |
|  | 119 | +5bp/-22bp | +1bp/-1bp | +1bp | +1bp/+1bp/-40bp |
|  | 120 | +1bp/-5bp | +1bp | +1bp | -39bp |
|  | 121 | -1bp/-2bp | +1bp/+1bp | +1bp/+11bp/WT | +1bp/-36bp |
|  | 123 | -3bp/-1bp | +1bp | -37bp | +6bp/+1bp/WT |
|  | 125 | +1bp/-1bp | +1bp/-2bp | +1bp/-28bp | +1bp/-1bp |
|  | 129 | -2bp/-32bp | +1bp/-13bp | -11bp | +18bp/+1bp/+2bp |
|  | 129 (T1 generation) | -2bp/-32bp | +1bp/-13bp | -11bp | +18bp/+2bp |
|  | 131 | -2bp | -13bp | +1bp/+1bp/-1bp | +1bp/+2bp |
|  | 137 | -1bp/-20bp | +1bp/-1bp | +1/+1/-1/-2/(-66+4) | +1bp/(-3+9)bp |
|  | 137 (T1 generation) | -1bp/-20bp | +1bp/-1bp | many* | +1bp/(-3+9)bp |
|  | 138 | -1 | +1/+1 | +1/+1 | +1/+2 |
|  | 140 | +1bp/-3bp | +1bp/+1bp | +1bp/-1bp | +1bp/+2bp/+2bp/-6bp |
|  | 140 (T1 generation) | +1bp/-3bp | +1bp/+1bp | +1bp/-1bp | +1bp/-6bp |
|  | 143 | -3bp/-10bp | +1bp/-1bp | (-2+5)bp/+1bp | +1bp/+2bp/-1bp |
|  | 143 (T1 generation) | -3bp/-10bp | +1bp/-1bp | -12bp/+1bp | +1bp/+2bp/-1bp |
|  | 149 | -3bp/-19bp | +1bp/+1bp/-43bp | -12bp | +1bp/+2bp |
|  | 151 | -2bp/-3bp | +1bp/+1bp | +1bp/-1bp/-30bp | +1bp/+2bp |
|  | 157 | -1bp/-1bp/-2bp | +1bp/-16bp | +1bp/-1bp/-27bp | SNP/+1bp/+2bp |
| Tx623/Macia SRH | 3 | +1bp/-8bp | +1bp/-1bp | -2bp | (-11+50)bp/WT |
|  | 5 | -1bp/-2bp | +1bp/+1bp | (-10+1)bp/+1bp | +1bp/-15bp/WT |
|  | 19 | -3bp/-9bp | -13bp | +1bp/-27bp | +1bp/-12bp/+2bp |
|  | 21 | -13bp | +1bp/-13bp | -1bp | +1bp/+2bp |
|  | 22 | -3bp/-5bp | +1bp | -26bp | -3bp |
|  | 25 | -1bp/-1bp | +1bp/-7bp | +1bp/+1bp/WT | +1bp/-6bp |
|  | 33 | -21bp/-25bp | +1bp/-1bp | -4bp | +6bp/WT |
|  | 36 | -22bp | +1bp/-13bp | -45bp/-30bp | -23bp/(-9+22)bp |
|  | 38 | -8bp/-15bp | +1bp/+1bp | -1bp/-5bp/-19bp/+1bp/-60bp | +1bp/-23bp |
|  | 39 | -3bp/-5bp | +1bp/+1bp | -1bp/+3bp | -35bp/-39bp |
|  | 52 | -8bp | +1bp/-1bp | -1bp/+1bp | +1bp/-3bp |
|  | 55 | -2bp/-55bp | +1bp/+1bp | +1bp/(-40+30)bp | +1bp/(-10+1)bp |
|  | 60 | -1bp/-1bp | +1bp/+1bp | -19bp | -6bp/-35bp |
|  | 63 | -1bp/-3bp | +1bp/+2bp | +1bp/-6bp | +1bp/-1bp |
|  | 64 | -3bp/-4bp | +1bp/-1bp | -1bp/-11bp | +2bp/-6bp |
|  | 65 | +1bp/-2bp | +1bp/+1bp | -9bp/-31bp | +2bp/-1bp |
|  | 72 | +1bp/+1bp/+1bp | +1bp | +1bp/-3bp/-62bp | +1bp/-12bp |
|  | 78 | +1bp/-2bp | +1bp/+1bp | (-55+9)bp/(-74+13)bp | +1bp/SNP |
|  | 88 | -3bp/-55bp | +1bp | -12bp | +1bp/+2bp/-24bp |
|  | 90 | -2bp/-6bp | -1bp/+1bp | -1bp/+1bp | +1bp/+2bp |
|  | 91 | -3bp/+20bp | +2bp/-7bp | -1bp | -12bp |
|  | 92 | -1bp/+1bp | +1bp/+1bp | -1bp/+1bp/-29bp/-6bp | +1bp/+1bp/+2bp |
|  | 94 | +1bp/-2bp | +1bp/-7bp | +1bp | -3bp/-52bp |
|  | 96 | -22bp/-45bp/22bp/+1bp/-45bp/-45bp | +1bp/+1bp | -7bp/-3bp | +1bp/+2bp |
|  | 97 | -3bp/+1bp | -1bp/-2bp | -30bp | +2bp |
|  | 98 | -8bp/-25bp | +1bp/+1bp | -7bp/(-19+32)bp | -15bp/+2bp/(-14+1)bp |
|  | 99 | -1bp/+1bp | +1bp/+1bp | +1bp/-5bp/-23bp/SNP | +1bp/-12bp |
|  | 100 | -1bp | +1bp/-1bp | +2bp/-57bp | +2bp/-1bp/(+3-1)bp |
|  | 102 | +1bp/-1bp | +1bp/-1bp | (-4+22)bp | +1bp |
|  | 105 | +1bp/-1bp | +1bp/+1bp | -11bp | +1bp/-9bp |
|  | 109 | -1bp/-5bp | +1bp/-13bp | -30bp | -1bp |
|  | 110 | -1bp/-1bp | +1bp/+1bp | -7bp/-24bp | +1bp/+2bp/+2bp |
|  | 114 | -2bp/-6bp | +1bp/-1bp | +1bp/-18bp | -1bp/-16bp |
|  | 115 | +1bp/-2bp | +1bp/+1bp | -11bp | +1bp/(-20+9)bp |
|  | 116 | -2bp/-2bp | +1bp/+1bp | -2bp | +1bp/+2bp/+2bp |
|  | 120 | -1bp/+2bp | +1bp | -1bp | +1bp/(-10+1)bp |
|  | 125 | -2bp/-22bp/-32bp | +1bp/+1bp | +1bp/-8bp | +1bp/+1bp |
|  | 127 | -2bp/-5bp | +1bp/+1bp | -2bp | +1bp/-1bp |
|  | 129 | +1bp/-5bp | +1bp/+1bp | -3bp | +1bp/(+10-10)bp |
|  | 144 | -3bp/-22bp | +1bp | -5bp/-18bp | +2bp/-9bp |
|  | 147 | -2bp/-6bp | +1bp | +1bp/-43bp | +1bp/-6bp |
|  | 154 | -2bp/-6bp | +1bp | (-26+3)bp/-8bp | +1bp/-1bp |
|  | 157 | -3bp/-4bp | +1bp/+1bp | +2bp/-1bp | +2bp/-3bp |
|  | 160 | -8bp/-13bp | +1bp/-8bp | +1bp/-4bp | -14bp/(-22+4)bp |

Supplemental Table 6. Primers sequences used for amplicon analysis of CRISPR/Cas9 edits

| Primer name | Sequence |
| --- | --- |
| *Spo11-1* gRNA1-FP | AGCCCAGTTCTGTTCCTTCG |
| *Spo11-1* gRNA1-RP | CAACGGAACCTTTGATCCTG |
| *Spo11-1* gRNA2-FP | TGCATGTATCTTGGCTCACTG |
| *Spo11-1* gRNA2-RP | CATTGAGATTGTGCCGACTG |
| *Rec8* gRNA1-FP | CGTTTAGTGAGGAGATTTTGAACC |
| *Rec8* gRNA1-RP | TTTCGATCACGGGGAATAAA |
| *Rec8* gRNA2-FP | CGTGCAGACTGAGATCAACG |
| *Rec8* gRNA2-RP | AAACGCTAAGACAGGGATGG |
| *OsdL1* gRNA1-FP | TGTCACAGTCAAGCAACAAGG |
| *OsdL1* gRNA1-RP | CACGGAATTCGGAGAAGAACG |
| *OsdL1* gRNA2-FP | ATGTACAAACCGCAACATGC |
| *OsdL1* gRNA2-FP | CCTCAATTGTTGGCACACC |
| *OsdL3* gRNA1-FP | TCACTGTCTCACCGATATGC |
| *OsdL3* gRNA1-RP | CCCTCCTCCTGATAAAAATGC |
| *OsdL3* gRNA2-FP | CTTAGCCGCCAGTCAAAGG |
| *OsdL3* gRNA2-FP | GAAGCCAACTGAAGTTGTAGTGC |
